# Who benefits from individual placement and support? A meta-analysis

**DOI:** 10.1017/S2045796022000300

**Published:** 2022-07-11

**Authors:** Lars de Winter, Chrisje Couwenbergh, Jaap van Weeghel, Sarita Sanches, Harry Michon, Gary R. Bond

**Affiliations:** 1 Phrenos Center of Expertise for Severe Mental Illnesses, Utrecht, the Netherlands; 2 Movisie Netherlands Centre for Social Development, Utrecht, the Netherlands; 3 Westat, Lebanon, NH, USA

**Keywords:** Mental health, randomised controlled trials, rehabilitation, systematic reviews

## Abstract

**Aims:**

Individual placement and support (IPS) is an evidence-based service model to support people with mental disorders in obtaining and sustaining competitive employment. IPS is increasingly offered to a broad variety of service users. In this meta-analysis we analysed the relative effectiveness of IPS for different subgroups of service users both based on the diagnosis and defined by a range of clinical, functional and personal characteristics.

**Methods:**

We included randomised controlled trials that evaluated IPS for service users diagnosed with any mental disorder. We examined effect sizes for the between-group differences at follow-up for three outcome measures (employment rate, job duration and wages), controlling for methodological confounders (type of control group, follow-up duration and geographic region). Using sensitivity analyses of subgroup differences, we analysed moderating effects of the following diagnostic, clinical, functional and personal characteristics: severe mental illness (SMI), common mental disorders (CMD), schizophrenia spectrum disorders, mood disorders, duration of illness, the severity of symptoms, level of functioning, age, comorbid alcohol and substance use, education level and employment history.

**Results:**

IPS is effective in improving employment outcomes compared to the control group in all subgroups, regardless of any methodological confounder. However, IPS was relatively more effective for service users with SMIs, schizophrenia spectrum disorders and a low symptom severity. Although IPS was still effective for people with CMD and with major depressive disorder, it was relatively less effective for these subgroups. IPS was equally effective after both a short and a long follow-up period. However, we found small, but clinically not meaningful, differences in effectiveness of IPS between active and passive control groups. Finally, IPS was relatively less effective in European studies compared to non-European studies, which could be explained by a potential benefits trap in high welfare countries.

**Conclusions:**

IPS is effective for all different subgroups, regardless of diagnostic, clinical, functional and personal characteristics. However, there might be a risk of false-positive subgroup outcomes and results should be handled with caution. Future research should focus on whether, and if so, how the IPS model should be adapted to better meet the vocational needs of people with CMD and higher symptom severity.

## Introduction

Employment is key to improve community functioning and mental health in people with mental illnesses (Drake and Wallach, 2020). Work fosters a sense of pride and self-esteem, offers financial independence, provides coping strategies for psychiatric symptoms and ultimately facilitates the process of recovery (Dunn *et al*., 2008). However, depending on the diagnosis, only between 14 and 33% of the working-age adults (18–65 years old) with mental illnesses are employed, which is substantially lower than the general population (Marwaha *et al*., 2007; Kozma *et al*., 2010; Hakulinen *et al*., 2020). Therefore, ongoing support in obtaining and sustaining competitive employment is needed for people with mental illnesses to create a strong and inclusive labour market. Individual placement and support (IPS) is the most effective rehabilitation programme to help people with mental illnesses into competitive employment (Modini *et al*., 2016; Metcalfe *et al*., 2018a).

IPS was originally developed to support people with severe mental illness (SMI) in achieving competitive employment. IPS is based on eight basic principles (Becker and Drake, 2003; See Box 1). The overall effectiveness of IPS is well-established for people with SMI (Modini *et al*., 2016; Metcalfe *et al*., 2018a). Because of its success, IPS is also increasingly offered to people with other diagnoses, such as common mental disorders (CMD), affective disorders, post-traumatic stress disorders (PTSD) and substance use disorders (SUD) (Bond *et al*., 2019). Results indicated the beneficial effects of IPS for people with PTSD and SUD (Bond *et al*., 2019). However, mixed indications of the effectiveness of IPS for people with CMD (including affective disorders) were found (Hellström *et al*., 2021; Probyn *et al*., 2021). The reasons for the diminished effectiveness of IPS for CMD are unclear, because a consistent definition of CMD is lacking. Diagnostic criteria for labelling CMD differ between studies, but most studies define CMD to include affective and/or anxiety disorders, of varying duration of illness (Vollebergh *et al*., 2001; Steel *et al*., 2014; De Vries *et al*., 2016).

Box 1. The eight basic principles of IPS (Becker and Drake, 2003)

**Table:** 

Principle	Explanation
1. Goal of competitive employment	The goal of IPS is obtaining and sustaining competitive employment. This is defined as jobs anyone can apply for, pay at least minimum wage/same pay as coworkers with similar duties, and have no artificial time limits imposed by the social service agency.
2. Zero exclusion and eligibility based on client choice	People are not excluded on the basis of readiness, diagnoses, symptoms, substance use history, psychiatric hospitalisations, homelessness, level of disability or legal system involvement.
3. Attention to client preferences	IPS programme services are based on each job seeker's preferences and choices rather than the employment specialist's and supervisor's judgments.
4. Rapid job search	IPS programmes use a rapid job search approach to help job seekers obtain jobs rather than assessments, training, & counselling. The first face to face contact with the employer occurs within 30 days.
5. Integration with mental health treatment	IPS programmes are integrated with mental health treatment teams. Employment specialists attach to 1 or 2 mental health treatment teams, which discuss their caseload.
6. Personalised benefits counselling	Employment specialists help people obtain personalised, understandable, and accurate information about their Social Security, Medicaid and other government entitlements.
7. Targeted job development	Employment specialists systematically visit employers, who are selected based on the job seeker's preferences, to learn about their business needs and hiring preferences.
8. Individualised, long-term support	Job supports are individualised and continue for as long as each worker wants and needs the support. Employment Specialists have face to face contact at least monthly.

Employment outcomes in IPS programmes also vary between service users with different clinical, functional and personal characteristics, such as symptom severity, substance use, involuntary hospitalisation, social functioning, work experience, education level, duration of illness, age and age of onset of psychiatric disorder (Catty *et al*., 2008; Marwaha *et al*., 2009; Campbell *et al*., 2010; Luciano *et al*., 2014; Fyhn *et al*., 2020; Christensen *et al*., 2021). However, these moderating effects have been inconsistent across studies, resulting in ambiguity about the effectiveness of IPS in different subgroups. As IPS has been increasingly expanded to different populations, it is timely to investigate how well the effectiveness of IPS generalises to new target groups.

Therefore, in this meta-analysis we analysed the relative effectiveness of IPS for different subgroups of service users as reported in randomised controlled trials of IPS. We assessed the relative effectiveness of IPS by examining study-level outcomes for subgroups of studies with different diagnostic, clinical, functional and personal characteristics using sensitivity analyses of subgroup differences (Borenstein and Higgins, 2013). This is the first meta-analysis that specifically focused on the relative effectiveness of IPS in different target groups with a focus on both target groups with and without SMI. This gives some unique insights into the relative effectiveness of IPS, and valuable addition to the recent contributions about this topic in comparable reviews (i.e. Bond *et al*., 2019; Probyn *et al*., 2021). The meta-analysis addressed the following research questions:

1. How does the effectiveness of IPS differ between subgroups of service users with distinct clinical, functional and personal characteristics?
2. What is the relative effectiveness of IPS for specific diagnostic subgroups of service users with CMD, SMI, schizophrenia spectrum disorders and mood disorders?

## Materials and methods

Our meta-analysis followed the latest PRISMA guidelines (Page *et al*., 2021). Our protocol was preregistered in PROSPERO (CRD42020220080).

### Search strategy

We identified records through searches in PubMed, PsycInfo and Cochrane of peer-reviewed journals until July 2019. The search was based on terms related to specific primary diagnoses (e.g., schizophrenia, mood disorder, anxiety disorder, but also CMD and SMI), IPS and other vocational rehabilitation programmes and competitive employment (see online Supplementary materials 1). We found additional references through reference lists of identified studies and systematic reviews.

### Study selection process

The included studies meet the following criteria:

#### Participant population

We included studies that investigated people who were diagnosed with any mental disorder, as determined by DSM-III to DSM-5 (American Psychiatric Association, 1997, 2000, 2013) or ICD 10–11 criteria (World Health Organization, 2016, 2019). Participants without mental disorders or at risk of developing mental health problems were excluded from the meta-analysis.

#### Study design

We included all randomised controlled trials that evaluated the effectiveness of IPS compared to at least one control condition in the meta-analysis.

#### Intervention

We included studies investigating a treatment arm comprised of IPS as a stand-alone intervention, not augmented with another active intervention, such as cognitive remediation or social skills training, confirmed by an IPS fidelity assessment, receiving at least ‘fair’ fidelity. For studies investigating the effectiveness of both IPS and IPS augmented with another intervention, we only included the IPS-only arm in the analyses and excluded the IPS augmented with another intervention- arm from the analysis.

#### Comparison

The control group could be any other vocational service or a passive control group (i.e., service as usual or waiting list).

#### Outcomes

The study reported competitive employment outcomes.

Two authors (LdW & CC) independently executed study selection, including both title and abstract screening and full-text screening. Disagreements of the full-text selection process were resolved by consensus.

### Data extraction

We extracted study details, participant characteristics, treatment variable, outcomes and study design data from all studies in this meta-analysis. Author LdW executed data extraction and discussed and resolved uncertainties with CC. Details about the data-extraction are presented in online Supplementary materials 2.

### Data synthesis

#### Assessment of outcomes

The included studies reported a variety of competitive employment outcomes. Therefore, we focused on three outcome measures of competitive employment that were reported by at least ten studies, the minimum number to provide outcomes with sufficient statistical power in meta-analyses (Jackson and Turner, 2017; Borenstein *et al*., 2021): (1) Competitive employment rate (i.e., the proportion of participants competitively employed for at least one day during the study period); (2) Job duration (i.e., days, weeks or months competitive employed during the study period); (3) Wages (i.e., total earnings from competitive employment during the study period).

#### Assessment of study design and region

The included studies also differed in study design, which might affect outcomes: studies compared IPS with a variety of control groups and outcomes were analysed over different follow-up periods. Furthermore, previous research also indicated regional differences (i.e., European versus non-European studies) in the effectiveness of IPS (Drake *et al*., 2019). Therefore, we analysed the study outcomes within specific subgroups based on these three confounding factors as follows: (1) type of control group: studies with an active control group encompassing treatment as usual combined with any other vocational services versus studies with a passive control group with treatment as usual and no primary focus on improvement of vocational functioning; (2) follow-up duration: an assessment period of 12 months or less versus more than 12 months; (3) Region: European studies versus non-European studies.

#### Assessment of moderators of outcomes

In order to answer our two research questions, we selected moderators of study outcomes from the included studies. The selection was based on the identification of relevant moderators analysed in previous studies and the availability of extractable raw data of these moderators in at least ten of our included studies (Borenstein *et al*., 2021).

*Assessment of diagnostic subgroups***.** For this study, we assessed subgroups of SMI or CMD based on diagnosis, duration of illness and inclusion criteria of studies (see Table 1). These three criteria were partly based on previous literature (i.e., Steel *et al*., 2014; De Vries *et al*., 2016). However, due to the lack of a consistent definition of CMD, we pragmatically translated these criteria based on the availability of data in the included studies. If studies met none of the three criteria we labelled them as ‘unclear’ and did not include these studies in the analysis. We were also able to include SSD and major depressive disorder as separate moderators. We divided these moderators into subgroups of studies in which the majority (i.e., >50%) of the study sample was diagnosed with the specific diagnosis and subgroups of studies in which the minority was diagnosed with the specific disorder (see Table 1).

**Table 1.** Operationalisations of moderators

1. Diagnosis				
Moderator	Comparison	Assessment instruments (*N* studies implementing instrument)^a^	Operationalisation	Studies with specific moderator
Severity of illness	Severe mental illness (SMI) vs. common mental disorders (CMD)	1. Diagnosis (21) 2. Duration of illness (0) 3. Inclusion criteria (2)	(1) Diagnosis: SMI: at least 75% of the study sample schizophrenia spectrum or bipolar disorder, CMD: at least 75% of the study sample mood or anxiety disorder (2) Duration of illness: SMI :at least 50% of the study sample schizophrenia spectrum or bipolar disorder and duration of illness at least 2 years; CMD: none of the study sample schizophrenia spectrum or bipolar disorder and duration of illness less than 2 years (3) Inclusion criteria: SMI or CMD are specifically mentioned in the inclusion criteria	SMI (20 studies): Bejerholm 2014; Bond 2007; Bond 2015; Burns 2007; Christensen 2019; Drake 1996; Drake 1999; Erickson 2020; Gold 2006; Howard 2010; Killackey 2008; Killackey 2019; Latimer 2006; Lehman 2002; Mueser 2004; Reme 2019; Tsang 2009; Twamley 2012; Waghorn 2014; Zhang 2017 CMD (5 studies): Davis 2012; Davis 2018; Hellström 2017; Poremski 2015; Reme 2019
Schizophrenia spectrum disorder (SSD)	>50% SSD vs ⩽50% SSD	1. ICD-10 (4) 2. DSM-IV or -5 (6) 3. OPCRIT (1) 4. SCID (8) 5. MINI (3) 6. CAPS-IV (1) 7. SCAN (2) 8 Clinical records (1) 9. Unclear (5)	Percentage of the study sample diagnosed with a schizophrenia spectrum disorder	>50% SSD (20 studies): Bejerholm 2014; Bond 2007; Bond 2015; Burns 2007; Christensen 2019; Drake 1999; Erickson 2020; Gold 2006; Howard 2010; Killackey 2008; Killackey 2019; Latimer 2006; Lehman 2002; Michon 2014; Mueser 2004; Tsang 2009; Twamley 2012; Waghorn 2014; Wong 2008; Zhang 2017 ⩽50% SSD (11 studies): Bejerholm 2017; Davis 2012; Davis 2018; Drake 1996; Drake 2013; Hellström 2017; Hoffmann 2012; Lones 2017; Poremski 2015; Reme 2019; Viering 2015
Major Depressive Disorder (MDD)	>50% MDD vs ⩽50% MDD	1. ICD-10 (4) 2. DSM-IV or −5 (6) 3. OPCRIT (1) 4. SCID (8) 5. MINI (3) 6. CAPS-IV (1) 7. SCAN (2) 8 Clinical records (1) 9. Unclear (5)	Percentage of the study sample diagnosed with a major depressive disorder	>50% MDD (4 studies): Bejerholm 2017; Drake 2013; Hellström 2017; Poremski 2015 ⩽50% MDD (23 studies): Bond 2007; Bond 2015; Burns 2007; Christensen 2019; Davis 2012; Davis 2018; Drake 1996; Drake 1999; Erickson 2020; Gold 2006; Hellström 2017; Hoffmann 2012; Howard 2010; Killackey 2008; Killackey 2019; Lehman 2002; Lones 2017; Mueser 2004; Poremski 2015; Reme 2019; Twamley 2012; Waghorn 2014; Zhang 2017
2. Clinical, functional and personal characteristics				
Moderator	Comparison	Assessment instruments (N studies implementing instrument)	Operationalisation	Studies with specific moderator
Duration of illness at baseline	Long duration of illness vs. Short duration of illness	Not Applicable	We extracted the duration of illness at baseline from the included studies that reported this construct and we calculated the median duration of illness from all included studies (i.e. 11.5 years of illness). All studies with a duration of illness above the median duration of illness were clustered in the ‘long duration of illness’ group, all studies with a duration of illness below the median duration of illness were clustered in the ‘short duration of illness' group,	Long duration of illness (5 studies): Bejerholm 2017; Davis 2018; Lehman 2002; Oshima 2014; Twamley 2012 Short duration of illness (6 studies): Bejerholm 2014; Burns 2007; Hoffmann 2012; Killackey 2008; Reme 2019; Viering 2015
Baseline severity of symptoms	Low baseline severity of symptoms vs. High baseline severity of symptoms	1. BPRS (9) 2. DTS (1) 3. HADS (1) 4. HDRS (1) 5. MADRS (1) 6. MHI-5 (1) 7. PANSS (6) 8. PCL-5 (1) 9. SANS (2) 10. SF-12 mental health (1)	From all symptom scales that were reported in the included studies, we searched for studies that assessed the psychometric quality of each scale based on a comparable population (i.e. ‘reference group studies') with the included study that assessed the specific moderator. We used the reference group studies as a basis to calculate percentile scores of the baseline level of symptoms from our included studies. By calculating percentile scores for each assessment instrument, we achieved homogeneous moderators assessed in the same scale range. Finally, we calculated the median percentile score of the normative percentile scores in order to cluster all studies into low (i.e. below-median symptom severity) and high (i.e. above-median symptom severity) symptom severity groups.	Low symptom severity (12 studies): Bejerholm 2014; Bond 2007; Burns 2007; Drake 1996; Drake 1999; Gold 2006; Hoffmann 2012; Howard 2010; Killackey 2008; Latimer 2006; Waghorn 2014; Zhang 2017 High symptom severity (12 studies): Bejerholm 2017; Christensen 2019; Davis 2012; Davis 2018; Drake 2013; Erickson 2020; Hellström 2017; Killackey 2019; Michon 2014; Mueser 2004; Reme 2019; Twamley 2012
Baseline Level of Functioning (LOF)	Low baseline LOF vs High baseline LOF	1. GAF (5) 2. GAS (2) 3. Post-Traumatic Stress-Related Functional Inventory Score (1) 4. Personal and social performance scale (1) 5. SAS-II (1) 6. SOFAS (2) 7. UPSA (1) 8. WHO-DAS 2.0 (1)	From all functioning scales that were reported in the included studies, we searched for studies that assessed the psychometric quality of each scale based on a comparable population (i.e. ‘reference group studies') with the included study that assessed the specific outcome. We used the reference group studies as a basis to calculate percentile scores of the level of functioning (LOF) at baseline from our included studies. By calculating percentile scores for each assessment instrument, we achieved homogeneous moderators assessed in the same scale range. Finally, we calculated the median percentile score of the normative percentile scores in order to cluster all studies into low (i.e. below-median LOF) LOF and high (i.e. above-median LOF) LOF groups.	Low LOF (8 studies): Christensen 2019; Davis 2012; Davis 2018; Drake 1996; Drake 1999; Hellström 2017; Hoffmann 2012; Howard 2010; High LOF (7 studies): Killackey 2008; Killackey 2019; Latimer 2006; Mueser 2004; Reme 2019; Twamley 2012; Zhang 2017
Age	⩽ 25th percentile (34.63 years) vs ⩾ 75th percentile (41.05 years)	Not Applicable	Based on the median and IQR we assessed which study samples' mean age was equal to or below the 25th percentile and which study samples' mean age was equal to are higher than 75th percentile.	Age ⩽ 25th percentile (8 studies): Christensen 2019; Erickson 2020; Hoffmann 2012; Killackey 2008; Killackey 2019; Waghorn 2014; Wong 2008; Zhang 2017 Age >75th percentile (8 studies): Bond 2015; Davis 2018; Drake 2013; Lehman 2002; Mueser 2004; Poremski 2015; Twamley 2012; Viering 2015
Comorbid alcohol use	Low comorbid alcohol use vs High comorbid alcohol use	Percentage (%) comorbid alcohol use	We extracted the percentage participants with comorbid alcohol use at baseline from the included studies that reported this construct and we calculated the median percentage comorbid alcohol use from all included studies (i.e. 22%). All studies with a comorbid alcohol use above the median were clustered in the ‘high comorbid alcohol use’, all studies with a comorbid alcohol use below the median were clustered in the ‘low comorbid alcohol use’ group	High comorbid alcohol use (6 studies): Bond 2007; Davis 2012; Davis 2018; Lones 2017; Poremski 2015; Twamley 2012 Low comorbid alcohol use (6 studies): Bond 2015; Drake 1996; Drake 1999; Gold 2006; Latimer 2006; Mueser 2004
Comorbid substance use	Low comorbid substance use vs High comorbid substance use	Percentage (%) comorbid substance use	We extracted the percentage participants with comorbid substance use at baseline from the included studies that reported this construct and we calculated the median percentage comorbid substance use from all included studies (i.e. 22%). All studies with a comorbid substance use above the median were clustered in the ‘high comorbid substance use’, all studies with a comorbid substance use below the median were clustered in the ‘low comorbid substance use’ group	High comorbid substance use (8 studies): Bond 2015; Davis 2012; Killackey 2008; Killackey 2019; Lehman 2002; Lones 2017; Poremski 2015; Twamley 2012 Low comorbid substance use (7 studies): Bond 2007; Drake 1996; Drake 1999; Gold 2006; Hoffmann 2012; Latimer 2006; Mueser 2004
Work experience	High work experience vs Low work experience	1. Number of months worked past 5 years (8) 2. % worked in past 5 years (10)	Work experience was assessed by extracting the number of months that participants have worked in competitive employment the past 5 years or the percentage of participants that have worked in competitive employment the past 5 years. For both constructs we calculated the median and labelled the studies as ‘high work experience’ if the number or percentage was above the median and it is labelled as ‘low work experience’ if the number or percentage was below the median. The median number of months that participants have worked in competitive employment the past 5 years was 16.4 months, and the median percentage of participants that have worked in competitive employment the past 5 years was 49%. If one study assessed both the number of months and the percentage of participants that worked in the past 5 years and one construct scored above and the other construct scored below the median, we chose to assess the construct that differed largest from the median to label work experience for the particular study.	High work experience (8 studies): Bond 2015; Burns 2007; Davis 2012; Drake 1996; Gold 2006; Howard 2010; Lones 2017; Michon 2014 Low work experience (8 studies): Bejerholm 2014; Bond 2007; Christensen 2019; Drake 1999; Latimer 2006; Lehman 2002; Mueser 2004; Wong 2008
Education level	Low education level vs High education level	ISCED level of education	The percentage of participants with specific education levels was extracted from the included studies. As studies were executed from different countries and each country has a different structure of education levels, we chose to label each education level based on the ISCED levels of education and calculated the percentage of participants with an ISCED level 5 or higher (tertiary education) within each study. We calculated the median percentage (i.e. 36.7%) of participants with an ISCED level of 5 or higher and labelled the studies below the median as ‘low education level’ and the studies above the median as ‘high education level’.	Low education level (11 studies): Bejerholm 2017; Bond 2015; Christensen 2019; Gold 2006; Hellström 2017; Hoffmann 2012; Mueser 2004; Reme 2019; Tsang 2009; Wong 2008; Zhang 2017 High education level (10 studies): Bond 2007; Davis 2012; Davis 2018; Drake 1996; Drake 2013; Erickson 2020; Latimer 2006; Lones 2017; Poremski 2015; Waghorn 2014

a BPRS, Brief Psychiatric Rating Scale; CAPS-IV, Administered PTSD Scale for DSM-IV; DSM-IV or −5, Diagnostic and Statistical Manual 4 or 5; DTS, Davidson Trauma Scale; GAF, Global Assessment of Functioning; GAS, Global Assessment Scale; HADS, Hamilton Anxiety and Depression Scale; HDRS, Hamilton Depression Rating Scale; ICD-10, International Classification of Diseases – 10; MADRS, Montgomery-Åsberg Depression Self Rating Scale; MHI-5, Mental Health Inventory – 5; MINI, Mini-International Neuropsychiatric Interview; PANSS, Positive and Negative Symptom Scale; PCL-5, PTSD checklist for DSM-5; SANS, Scale for the Assessment of Negative Symptoms; SAS-II, Simpson Angus Scale – II; SCAN, Structured Clinical Assessment in Neuropsychiatry; SCID, The Structured Clinical Interview for DSM-5; SOFAS, Social and Occupational Functioning Assessment Scale; SF-12, Short Form Health Survey 12; UPSA, UCSD performance-based skills assessment; WHO-DAS 2.0, World Health Organization Disability Assessment Scale 2.0.

*Clinical, functional and personal characteristics***.** We identified eight other moderators of outcomes: duration of illness, the severity of symptoms, level of functioning, age, comorbid alcohol and substance use, education level and employment history. We assessed these moderators at baseline and operationalised those into subgroups. Subgroups were generally assessed based on the available data in the included studies in this meta-analysis, in order to achieve equally distributed subgroups. Criteria for the operationalisation into subgroups of each moderator are described in Table 1.

### Risk of bias assessment

We assessed the risk of bias for each study through the Cochrane Collaboration risk of the bias assessment tool (Higgins and Green, 2008). Potential bias (i.e., high, low or unclear) is assessed as a judgment for individual elements from five domains (selection, performance, attrition, reporting and other bias). Author LdW rated all studies and CC independently rated the risk of bias of 50% of all studies. The inter-rater reliability (Cohen's kappa; McHugh, 2012) was substantial (*κ* = 0.61; Landis and Koch, 1977) and disagreements were resolved through consensus.

### Statistical analysis

#### Meta-analytic procedure

Meta-analyses were conducted using RevMan 5.3 (The Nordic Cochrane Centre, 2014). We assessed the effectiveness of IPS by analysing differences between IPS and the control group over the study period by calculating the standardised mean difference (d) for continuous outcomes (i.e., job duration and wages) and the odds ratio (OR) for categorical outcomes (i.e., employment rate). For studies reporting multiple outcome assessments for related outcome measures, we pooled the effect sizes into an overall effect size. We used random-effects models, weighted by the method of inverse variance (Higgins and Green, 2008). The magnitude of effect sizes was assessed based on the criteria described by Chinn (2000). Statistical heterogeneity was assessed by calculating the *I^2^* statistic (Higgins and Thompson, 2002). We performed the overall meta-analysis within separate subgroups based on the type of control group, follow-up duration and geographical region. We controlled for the potential influence of these factors using an analysis of subgroup differences (Borenstein and Higgins, 2013). One study had both an active and passive control group (Mueser *et al*., 2004), and multiple studies both followed service users after ⩽ 12 months and >12 months of follow-up. For the overall meta-analysis we pooled the effect sizes for all control groups or follow-up assessments within the study into one overall effect size, but we analysed both effect sizes separately during the analysis of subgroup differences, controlling for methodological confounders.

#### Calculating moderating effects

We analysed moderating effects through a sensitivity analysis of subgroup differences (Borenstein and Higgins, 2013), in which we compared subgroup outcomes with high levels or presence of the moderator versus those with low levels or absence of the moderator (see Table 1). Furthermore, the positive or negative influence of specific subgroups on employment outcomes was assessed by investigating which subgroups' confidence intervals of treatment effect exceeded the upper (‘positive’ influence) or lower (‘negative’ influence) bound of the confidence interval of the overall effect size of treatment effect.

#### Outliers and publication bias

We addressed the potential influence of outliers (i.e., if the confidence interval [CI] of an individual study outcome exceeded the CI of the overall effect size) by comparing the overall effect size of the outcome, including the outliers, with the overall effect size when outliers are removed through an analysis of subgroup differences. Potential publication bias was detected by visual inspection of funnel plots.

## Results

### Study flow

**Fig. 1.**
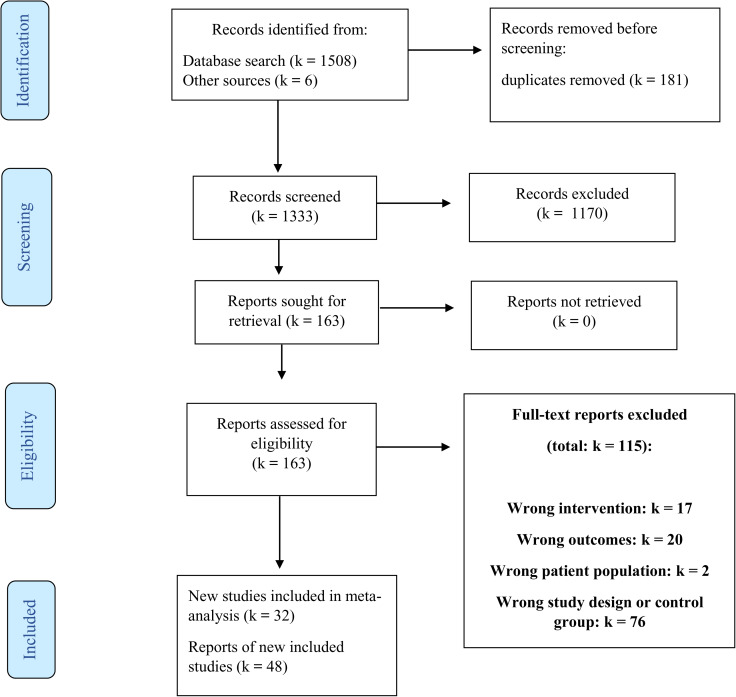
Flow chart selection studies conform Prisma guidelines.

Of the 1333 records retrieved through database search and reference tracking, 1170 records were excluded after the title and abstract screening. Of the remaining 163 reports, 115 reports were excluded after full-text selection (see Fig. 1 for reasons of exclusion). The remaining 48 reports reported the results of 32 studies.

### Study characteristics

As shown in Table 2, the 32 studies included 3818 participants receiving IPS and 3847 participants receiving a control intervention. The mean age of the aggregated sample (*n* = 7665) was 38.9 years (study range: 20.4–51.0); 44.1% of the participants were female. A total of 3454 (45.1%) participants were diagnosed with a schizophrenia spectrum disorder (SSD), and 2587 (33.8%) had a main diagnosis of major depressive disorder (MDD). The remaining 1624 (21.2%) had other diagnoses, such as anxiety disorder, PTSD, SUD or personality disorders. Twenty studies met the criteria for SMI and five studies met the criteria for CMD.

**Table 2.** Characteristics of included studies

Study^^a^^	*N* (IPS versus control(s))	Comparison group^b^	*N* sites	Country	Follow-up assessment	Study attrition	Age (M(s.d.))	Primary diagnosis	IPS fidelity score^b^	Outcomes
1. Bejerholm 2014^A,B^	**60**–60	Traditional vocational rehabilitation (A)	6	Sweden	18 months	27.50%	38.0 (8.0)	1. Schizophrenia and other psychosis: 64.7% 2. Bipolar disorder: 7.6% 3. Other diagnoses: 27.7%	IPS 25-item scale^c^: 6 months: 110 (G) 12 months: 115 (E) 18 months: 117 (E)	1. Competitive employment rate 2. Job duration
2. Bejerholm 2017^C^	**33**–28	Traditional vocational rehabilitation (A)	4	Sweden	6 & 12 months	4.90%	41.0 (11.0)	1. Depression: 68.9% 2. Bipolar disorder: 31.2%	IPS 25-item scale^c^: 12 months: 106 (G)	1. Competitive employment rate 2. Job duration
3. Bond 2007^D,E^	**96–**98	Diversified placement approach (A)	2	USA	24 months	25.30%	38.8 (9.6)	1. Schizophrenia: 39.0% 2. Schizoaffective disorder: 17.1% 3. Depression: 17.1% 4. Bipolar disorder: 24.1% 5. Other diagnoses: 2.7%	IPS 15-item scale^c^: Endpoint: 72 (E)	1. Competitive employment rate 2. Job duration
4. Bond 2015^F^	**45**–45	Work Choice (A)	2	USA	12 months	5.60%	43.8 (11.5)	1. Schizophrenia: 52.9% 2. Depression: 18.4% 3. Bipolar: 25.3% 4. Other diagnoses: 3.5%	IPS 25-item scale^c^: Good fidelity (exact scores not reported)	1. Competitive employment rate 2. Job duration
5. Burns 2007^G,H,I^	**156**–156	Vocational service (A)	6	Bulgaria / Germany/ Italy/ Netherlands/ Switzerland/ UK	18 months	19.20%	37.8 (9.9)	1. Schizophrenia /schizoaffective disorder: 80.3% 2. Bipolar disorder: 16.5% 3. Other psychotic disorders: 3.2%	IPS 15-item scale^c^: Endpoint: 65 (G)	1. Competitive employment rate 2. Job duration
6. Christensen 2019^K^	**243**–239	SAU (vocational rehabilitation) (A)	5	Denmark	18 months	27.00%	33.1 (10.1)	1. Schizophrenia spectrum disorder: 76.8% 2. Bipolar disorder: 11.8% 3. Recurrent depression: 11.4%	IPS 25-item scale^c^: Fidelity score range between IPS programmes: 75–101 (fair to good fidelity)	1. Competitive employment rate 2. Job duration 3. Wages
7. Davis 2012^M,N^	**42**–43	Standard VA Vocational Rehabilitation Programme (A)	1	USA	12 months	16.50%	40.2 (12.1)	Post-traumatic stress disorder: 100%	IPS 15-item scale^c^: Study period: 61 (F)	1. Competitive employment rate 2. Job duration 3. Wages
8. Davis 2018^O,P^	**271**–270	Transitional work programme (A)	12	USA	2.3; 4.6; 6.9; 9.2; 11.5; 13.8; 16.2 & 18 months	19.20%	42.2 (11.0)	Post-traumatic stress disorder: 100%	IPS 15-item scale^c^: Study period: 63–69 (Fair to Good)	1. Competitive employment rate 2. Job duration 3. Wages
9. Drake 1996^J,L^	**74**–69	Group Skills Training (A)	2	USA	18 months	2.10%	37.0 (9.5)	1. Schizophrenia or a related psychotic disorder: 46.9% 2. bipolar or other severe mood disorder: 42.7% 3. Other diagnoses: 10.5%	Fidelity scale and assessment score unclear and not reported	1. Competitive employment rate 2. Job duration 3. Wages
10. Drake 1999^Q,R^	**76**–76	Enhanced vocational rehabilitation (A)	2	USA	18 months	1.30%	39.4 (7.1)	1. Schizophrenia spectrum disorder: 67.1% 2. Bipolar disorder: 13.8% 3. Depressive disorder: 16.5% 4. Other Axis I disorder: 2.6%	IPS 15-item scale^c^: Exact scores and ratings not reported	1. Competitive employment rate 2. Job duration 3. Wages
11. Drake 2013^S,T^	**1121**–1117	Care as usual (P)	23	USA	18 & 24 months	8.20%	43.5 (NR^b^)	1. Schizophrenia: 29.7% 2. Affective disorder: 70.3%	IPS 15-item scale^c^: Percentage of IPS programmes with good fidelity: Year 1: 77% Year 2: 86% Year 3: 98%	1. Competitive employment rate 2. Job duration 3. Wages
12. Erickson 2021^U^	**56**–53	Treatment as Usual (P)	12	Canada	6 & 12 months	9.20%	23.1 (3.4)	1. Schizophrenia: 37.6% 2. Schizophreniform disorder: 4.6% 3. Schizoaffective disorder: 8.3% 4. Bipolar disorder: 18.4% 5. Major depression: 9.2% 6. Psychosis NOS: 15.6% 7. Substance-induced psychosis: 4.6% 8. Delusional disorder: 0.9% 9. Aspergers syndrome: 0.9%	IPS 25-item scale^c^: 1 year: 100 (G) 2 year: 110 (G)	1. Competitive employment rate 2. Job duration
13. Gold 2006^V^	**66**–77	Supported Employment Programme (A)	1	USA	24 months	24.50%	35.5 (NR^b^)	1. Schizophrenia spectrum disorder: 68.5% 2. Mood disorder: 31.5%	IPS 15-item scale^c^: Study period: 69 (G)	Competitive employment rate
14. Hellström 2017^W^	**162**–164	Job Centre services as usual (A)	NR^b^	Denmark	12 & 24 months	29.80%	35.0 (10.5)	1. Depression: 69.0% 2. Phobic anxiety: 7.7% 3. Other anxiety: 12.0% 4. Bipolar disorder: 11.4%	IPS-MA 21-item fidelity score: Score 102 out of 105	Job duration
15. Howard 2010^X,Y^	**109**–110	Treatment as Usual (P)	2	United Kingdom (UK)	12 & 24 months	13.70%	38.3 (9.4)	1. Psychotic disorder: 72.5% 2. Mood disorder: 27.5%	IPS 15-item scale^c^: Study period: 68 (G)	Competitive employment rate
16. Hoffmann 2012^Z,AA,AB^	**46**–54	Traditional vocational rehabilitation programmes (A)	1	Switzerland	24 & 60 months	12.00%	33.8 (9.4)	1. Schizophrenia spectrum disorder: 38.0% 2. Affective disorder: 41.0% 3. Other diagnosis: 21.0%	IPS 15-item scale^c^: Study period: 66–68 (G)	1. Competitive employment rate 2. Job duration 3. Wages
17. Killackey 2008^AC^	**20**–21	Treatment as Usual (P)	1	Australia	6 months	0.00%	21.4 (2.3)	Schizophrenia-spectrum disorder: 100%	IPS 15-item scale^c^: Study period: 68 (G)	1. Competitive employment rate 2. Job duration 3. Wages
18. Killackey 2019^AD^	**73**–73	Treatment as Usual (P)	1	Australia	18 months	13.00%	20.4 (2.4)	1. Schizophreni-form/schizophrenia: 43.8% 2. Schizoaffective disorder: 13.0% 3. Major depressive disorder, psychotic features: 11.6% 4. Bipolar disorder: 13.7% 5. Psychosis NOS: 11.6% 6. Other diagnoses: 6.2%	IPS 25-item scale^c^: Good fidelity (exact scores not reported)	Competitive employment rate
19. Latimer 2006^AE^	**75–**75	Usual services (P)	1	Canada	12 months	16.70%	40.2 (10.0)	1. Schizoaffective disorder: 16.8% 2. Other schizophrenia spectrum disorders: 59.1% 3. Bipolar disorder: 20.1% 4. Other diagnoses: 4.0%	IPS 15-item scale^c^: Study period: 71 (E)	1. Competitive employment rate 2. Job duration 3. Wages
20. Lehman 2002^AF^	**113**–116	Psychosocial rehabilitation programme (A)	1	USA	24 months	31.10%	41.5 (8.5)	1. Psychotic disorder: 78.3% 2. Mood disorders: 21.7%	IPS 15-item scale^c^: Study period: 69–71 (G)	1. Competitive employment rate 2. Wages
21. Lones 2017^AG^	**22**–23	Waitlist plus treatment as usual (P)	1	USA	6 & 12 months	22.20%	37.1 (10.6)	Moderate-to-severe opioid use disorder: 100%	IPS 25-item scale^c^: Study period: 85 (F)	1. Competitive employment rate 2. Job duration 3. Wages
22. Michon 2014^AH^	**71–**80	Traditional Vocational Rehabilitation (A)	4	Netherlands	6; 18 & 30 months	43.10%	34.9 (10.5)	1. Psychotic disorder: 54.3% 2. Other diagnoses: 45.7%	Quality of Supported Employment Implementation Scale (QSEIS): 2 IPS programmes Good fidelity; 2 IPS programmes Fair fidelity	Competitive employment rate
23. Mueser 2004^AI,AJ,AK^	**68**–67–69	1. Psychiatric Rehabilitation Centre (A) 2. Standard Services (P)	1	USA	24 months	18.60%	41.2 (9.2)	1. Schizophrenia: 53.4% 2. Schizoaffective disorder: 21.1% 3. Major depression: 17.2% 4. Bipolar disorder: 4.9% 5. Personality disorder: 1.0% Other diagnoses: 2.5%	IPS 15-item scale^c^: Study period: 71 (G)	1. Competitive employment rate 2. Job duration 3. Wages
24. Oshima 2014^AL^	**18**–19	Conventional vocational rehabilitation (A)	1	Japan	6 months	0.00%	40.6 (8.9)	NR^b^	IPS 15-item scale^c^: Study period: 68 (G)	1. Competitive employment rate 2. Job duration 3. Wages
25. Poremski 2017^AM^	**45**–45	Treatment as Usual (P)	1	Canada	8 months	5.60%	46.2 (10.0)	1. Major depressive disorder: 64.4% 2. Psychotic disorder: 22.2% 3. Panic disorder: 5.6% 4. Mania-hypomania: 4.4% 5. Post-traumatic stress disorder: 3.3%	IPS 25-item scale^c^: Study period: 100 (G)	1. Competitive employment rate 2. Wages
26. Reme 2019^AN^	**229**–181	High quality treatment as usual (A)	6	Norway	12 & 18 months	0.50%	35.0 (10.8)	1. Psychotic disorder: 27.1% 2. Bipolar disorder: 13.9% 3. Major depression: 40.0% 4. Anxiety disorder: 40.5% 5. Alcohol/drug abuse: 18.3% 6. Other diagnosis: 8.3%	IPS 25-item scale^c^: Good fidelity (exact scores not reported)	Competitive employment rate
27. Tsang 2009^AO,AP^	**65**–66	Traditional vocational rehabilitation (A)	5	Hong Kong	7; 11 & 15 months	31.80%	34.9 (8.5)	1. Schizophrenia: 76.7% 2. Other diagnoses: 23.3%	IPS 15-item scale^c^: Study period: 65–68 (G)	Competitive employment rate
28. Twamley 2012^A^^Q^	**30**–28	Conventional vocational rehabilitation (P)	1	USA	12 months	20.70%	51.0 (4.3)	1. Schizophrenia: 39.7% 2. Schizoaffective disorder: 60.3%	IPS 15-item scale^c^: Study period: 63 (F)	Competitive employment rate
29. Viering 2015^AR^	**127**–123	Other vocational services (P)	1	Switzerland	24 months	31.60%	42.6 (10.6)	1. Mood affective disorder: 47.2% 2. Schizophrenia/ schizoaffective disorder: 15.6% 3. Personality disorder: 17.2% 4. Other diagnoses: 18.0%	IPS 15-item scale^c^: Study period: 61 (F)	1. Competitive employment rate 2. Job duration
30. Waghorn 2014^AS^	**106**–102	Non-integrated forms of supported employment (A)	5	Australia	12 months	44.20%	32.4 (8.9)	1. Psychotic disorder: 80.8% 2. Bipolar disorder: 8.2% 3. Major depression or anxiety disorder: 6.3%	IPS 15-item scale^c^: Study period: 69 (G)	1. Competitive employment rate 2. Job duration
31. Wong 2008^AT^	**46**–46	Conventional vocational rehabilitation (A)	1	Hong Kong	6; 12 & 18 months	1.10%	33.6 (9.2)	1. Schizophrenia spectrum disorder; 69.6% 2. Affective disorder: 18.5% 3. Other diagnoses: 12.0%	IPS 15-item scale^c^: Study period: 69 (G)	1. Competitive employment rate 2. Job duration 3. Wages
32. Zhang 2017^AU^	**54**–54	Traditional Vocational Rehabilitation (P)	1	China	15 months	NR^b^	32.8 (8.3)	Schizophrenia: 100%	IPS 15-item scale^c^: Study period: 67 (G)	Competitive employment rate

a References of reports of included studies: A. Areberg and Bejerholm (2013); B. Bejerholm *et al*. (2015); C. Bejerholm *et al*. (2017); D. Bond *et al*. (2007); E. Bond *et al*. (2013); F. Bond *et al*. (2015); G. Burns *et al*. (2007); H. Burns and Cathy (2008); I. Kilian *et al*. (2012); J. Drake *et al*. (1996); K. Christensen *et al*. (2019); L. Clark *et al*. (1998); M. Davis *et al*. (2012); N. Davis *et al*. (2014); O. Davis *et al*. (2018*a*); P. Davis *et al*. (2018*b*); Q. Drake *et al*. (1999); R. Dixon *et al*. (2002); S. Drake *et al*. (2013); T. Metcalfe *et al*. (2018*b*); U. Erickson *et al*. (2021); V. Gold *et al*. (2006); W. Hellström *et al*. (2017); X. Howard *et al*. (2010); Y. Heslin *et al*. (2011); Z. Hoffmann *et al*. (2012); AA. Hoffmann *et al*. (2014); AB. Jäckel *et al*. (2017); AC. Killackey *et al*. (2008); AD. Killackey *et al*. (2019); AE. Latimer *et al*. (2006); AF. Lehman *et al*. (2002); AG. Lones *et al*. (2017); AH. Michon *et al*. (2014); AI. Mueser *et al*. (2001); AJ. Mueser *et al*. (2004); AK. Mueser *et al*. (2014); AL. Oshima *et al*. (2014); AM. Poremski *et al*. (2017); AN. Reme *et al*. (2019); AO. Tsang *et al*. (2009); AP. Tsang *et al*. (2011); AQ. Twamley *et al*. (2012); AR. Viering *et al*. (2015); AS. Waghorn *et al*. (2014); AT. Wong *et al*. (2008); AU. Zhang *et al*. (2017)

b A, active control group; E, excellent fidelity; F, Fair fidelity; G, good fidelity; NR, Not Reported; P, Passive control group;.

c IPS-15 item scale (Bond et al., 1997): item scale range: 15–75; Fidelity ratings: <55 = No IPS; 56–65 = Fair fidelity (F); >65 = Good fidelity (G); IPS-25 item scale (Bond, Peterson, Becker and Drake, 2012): item scale range: 25–125; Fidelity ratings: <74 = No IPS; 74–99 = Fair fidelity (F); 100–114 = Good fidelity (G); 115–125 = Exemplary fidelity (E).

Twenty-one studies compared IPS with an active control group and 12 studies compared IPS with a passive control group (including one study with both a passive and active control group). The overall study attrition rate (i.e., lost to follow-up) was 16.3% and only two studies reached a ‘high’ attrition rate exceeding 40%. Instruments for fidelity assessment differed between studies (see Table 2), but the majority of the studies (75.0%) achieved at least ‘good’ IPS programme fidelity.

### Quality assessment

**Fig. 2.**
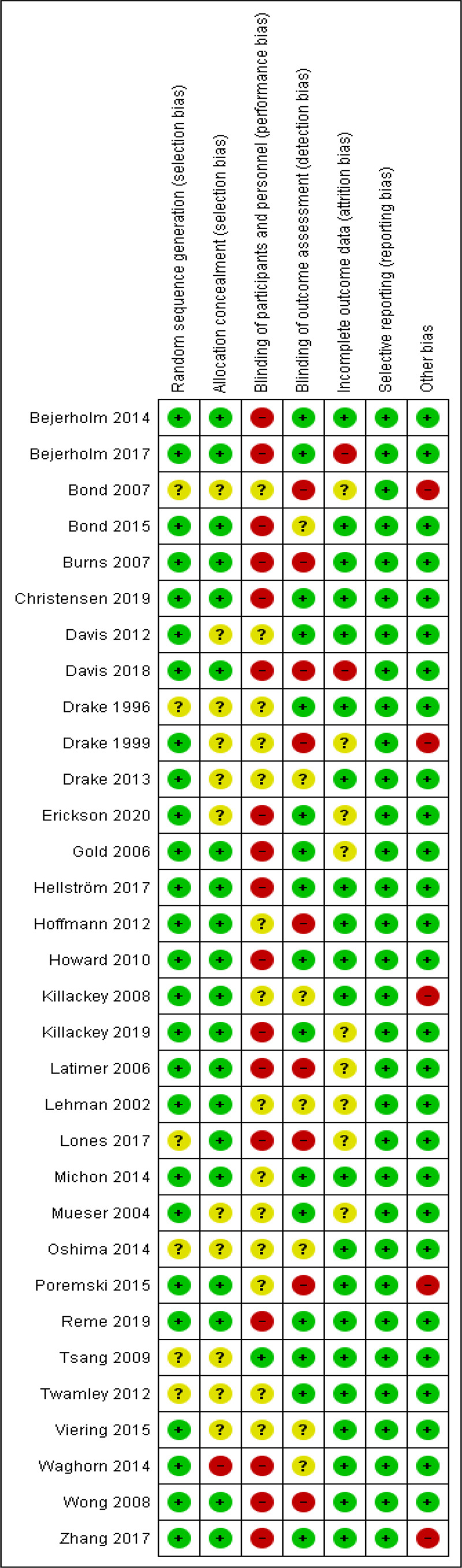
Cochrane risk of bias assessment.

Quality assessment is reported in Fig. 2. Overall we found low levels of selection and attrition bias, but relatively higher levels of performance and detection bias. The majority of studies (81.3%) reported a low risk of selection bias (i.e., random sequence generation and allocation concealment). In the majority of studies (53.1%) the participants and personnel were not blinded or information about blinding was unclear (43.4%). However, given the nature of the intervention and the study design, it was generally not feasible to achieve proper blinding of participants, so a certain level of performance bias was inevitable. In nine studies (29.0%) the outcome assessors were not blinded, indicating a high risk of detection bias. This is a relatively large number of studies with a high risk of detection bias, compared with the other risk of bias domains. However, the outcomes we used in our meta-analysis (i.e., employment rate, job duration and wages) are objective outcome measures and not sensitive for the interpretation of the outcome assessor. Therefore, this might not have a large influence on the study outcomes. In most studies (67.7%) we found a low risk of attrition bias (i.e., incomplete outcome data). Five studies reported other sources of bias: three studies reported baseline differences between IPS and the control group that potentially influenced outcomes and one study indicated the potential influence of allegiance bias because specialists favoured one intervention over the other and one study had a low fidelity score during the first part of the study which may have negatively influenced study outcomes at the start of the study. There were no indications of selective outcome reporting in any of the 32 studies.

### Overall meta-analysis

Thirty-one studies reported employment rate outcomes (see Table 3). A higher percentage of IPS participants (48.8%) than control group participants (28.3%) were employed during follow-up, showing small effect sizes (*OR*  =  2.62 [2.37–2.89], *p* < 0.01). Outcomes were moderately heterogeneous (*I^2^* = 74% [67–80%]; *p* < 0.01). The overall effect sizes of the employment rate were not influenced by the follow-up duration. However, we did find more favourable employment rate outcomes for IPS in non-European studies compared with European studies (*χ^2^* = 10.54; *p* < 0.01) and in studies that compared IPS with an active control group compared with a passive control group (*χ^2^* = 10.77; *p* < 0.01).

**Table 3.** Overall meta-analysis of outcomes

Employment rate												
Comparison	Follow-up (FU) subgroup	*N* studies^a^	% employed at FU^b^				Effect size of outcome^c^			Heterogeneity		
			IPS		Control		OR^d^	95% CI	*p*	*I*^2^	95% CI	*p*
			*n* (%)	*N*	*n* (%)	*N*						
IPS vs. active control condition	All studies	20	949 (50.1%)	1893	496 (26.7%)	1859	3.15 [S]	2.74–3.62	<0.01	79%	71–84%	<0.01
	⩽ 12 month FU	10	353 (38.3%)	921	182 (20.8%)	876	2.50 [S]	2.07–3.02	<0.01	68%	49–80%	<0.01
	> 12 month FU	15	895 (54.2%)	1652	518 (31.9%)	1625	2.88 [S]	2.48–3.36	<0.01	81%	72–87%	<0.01
	European studies (including UK)	7	345 (42.2%)	817	199 (25.5%)	781	2.27 [S]	1.84–2.79	<0.01	62%	32–79%	<0.01
	Non-European studies	13	604 (56.1%)	1076	297 (27.6%)	1077	4.09 [M]	3.40–4.92	<0.01	79%	68–86%	<0.01
IPS vs. passive control condition	All studies	12	846 (48.3%)	1753	525 (29.4%)	1788	2.26 [S]	1.97–2.68	<0.01	61%	43–74%	<0.01
	⩽ 12 month FU	7	144 (42.5%)	339	80 (23.6%)	338	2.12 [S]	1.52–2.96	<0.01	65%	37–81%	<0.01
	> 12 month FU	6	719 (50.8%)	1414	454 (31.3%)	1450	2.30 [S]	1.97–2.68	<0.01	55%	17–76%	0.05
	European studies (including UK)	3	112 (30.3%)	370	78 (21.3%)	367	1.64 [S]	1.18–2.27	<0.01	34%	0–74%	0.22
	Non-European studies	9	734 (53.1%)	1383	447 (31.5%)	1421	2.44 [S]	2.09–2.85	<0.01	62%	38–76%	<0.01
*Overall outcomes*		31	1745 (48.8%)	3578	1013 (28.3%)	3578	2.62 [S]	2.37–2.89	<0.01	74%	67–80%	<0.01
Test for subgroup differences		Active vs passive control condition						*χ*^2^ = 10.77; df = 1; *p* < 0.01				
		⩽ 12 month vs. > 12 month follow-up						*χ*^2^ = 0.29; df = 1; *p* = 0.59				
		European vs. non-European studies						*χ*^2^ = 10.54; df = 1; *p* < 0.01				
Job duration												
Comparison	Follow-up subgroup		M (s.d.) at FU^b^				Effect size of outcome^c^			Heterogeneity		
			IPS		Control							
		*N* studies^a^	M (s.d.)	*N*	M (s.d.)	*N*	*d*^d^	95% CI	*p*	*I*^2^	95% CI	*p*
IPS vs. active control condition	All studies	17	24.5 (11.4)	1571	10.9 (10.0)	1565	0.47 [S]	0.33–0.61	<0.01	81%	73–87%	<0.01
	⩽ 12 month follow-up	7	27.1 (13.6)	674	15.2 (13.9)	666	0.42 [S]	0.16–0.68	<0.01	76%	51–88%	<0.01
	> 12 month follow-up	10	22.7 (9.9)	897	7.9 (5.1)	899	0.47 [S]	0.30–0.63	<0.01	82%	73–89%	<0.01
	European studies	7	21.0 (12.2)	688	10.2 (8.0)	694	0.40 [S]	0.20–0.61	<0.01	83%	68–91%	<0.01
	Non-European studies	10	27.8 (10.8)	832	12.4 (11.5)	828	0.52 [M]	0.33–0.71	<0.01	77%	62–86%	<0.01
IPS vs. passive control condition	All studies	7	27.1 (12.7)	1354	17.1 (13.7)	1393	0.31 [S]	0.12–0.49	<0.01	69%	42–83%	<0.01
	⩽ 12 month follow-up	4	26.4 (17.9)	155	18.0 (16.9)	152	0.23 [S]	0.07–0.40	<0.01	0%	0–78%	0.63
	> 12 month follow-up	3	28.1 (1.9)	1199	16.0 (11.3)	1241	0.36 [S]	0.02–0.71	<0.05	87%	56–96%	<0.01
	European studies	1	29.6 (19)	127	27.7 (19.5)	121	−0.02 [N]	−0.27 to 0.23	0.88	NA	NA	NA
	Non-European studies	6	26.7 (13.9)	1227	15.4 (14.1)	1272	0.37 [S]	0.20–0.54	<0.01	54%	15–75%	0.06
*Overall outcomes*		23	25.2 (11.8)	2857	13.1 (11.4)	2889	0.41 [S]	0.30–0.52	<0.01	77%	69–83%	<0.01
Test for subgroup differences		Active vs passive control condition						*χ*^2^ = 1.98; df = 1; *p* = 0.16				
		⩽ 12 month vs. > 12 month follow-up						*χ*^2^ = 0.27; df = 1; *p* = 0.60				
		European vs. non-European studies						*χ*^2^ = 0.65; df = 1; *p* = 0.42				
Wages												
Comparison	Follow-up subgroup		M (s.d.) at FU^b^				Effect size of outcome^c^			Heterogeneity		
			IPS		Control							
		*N* studies^a^	M (s.d.)	*N*	M (s.d.)	*N*	*d*^d^	95% CI	*p*	*I*^2^	95% CI	*p*
IPS vs. active control condition	All studies	10	286.6 (314.7)	994	148.3 (196.6)	979	0.39 [S]	0.20–0.58	<0.01	76%	61–85%	<0.01
	⩽ 12 month follow-up	2	310.1 (328.5)	60	77.9 (94.6)	62	0.63 [M]	0.26–0.99	<0.01	0%	NA	0.92
	> 12 month follow-up	8	277.8 (332.2)	934	174.7 (223.0)	917	0.35 [S]	0.15–0.56	<0.01	80%	64–89%	<0.01
	European studies (including UK)	2	558.9 (491.9)	289	353.1 (247.2)	285	0.17 [N]	−0.07 to 0.41	0.17	52%	NA	0.15
	Non-European studies	8	226.1 (266.3)	705	102.8 (166.9)	694	0.46 [S]	0.22–0.69	<0.01	77%	59–87%	<0.01
IPS vs. passive control condition	All studies	6	497.7 (413.9)	1222	414.8 (401.1)	1259	0.28 [S]	0.14–0.42	<0.01	23%	0–43%	0.26
	⩽ 12 month follow-up	4	640.4 (451.7)	150	550.2 (427.3)	141	0.15 [N]	–0.07 to 0.36	0.19	0%	0–79%	0.61
	> 12 month follow-up	2	252.1 (252.4)	1072	144.1 (191.8)	1120	0.39 [S]	0.11–0.67	<0.01	64%	NA	0.09
	European studies (including UK)	0	X	X	X	X	X	X	X	X	X	X
	Non-European studies	6	497.7 (413.9)	1222	414.8 (401.1)	1261	0.28 [S]	0.14–0.42	<0.01	23%	0–43%	0.26
*Overall outcomes*		15	379.1 (358.9)	2148	257.0 (306.4)	2172	0.31 [S]	0.19–0.44	<0.01	65%	51–76%	0.01
Test for subgroup differences		Active vs passive control condition						*χ*^2^ = 0.84; df = 1; *p* = 0.36				
		⩽ 12 month vs. > 12 month follow-up						*χ*^2^ = 0.14; df = 1; *p* = 0.70				
		European vs. non-European studies						*χ*^2^ = 1.58; df = 1; *p* = 0.21				

a Some studies have used multiple follow-up assessments or have multiple treatment arms. Therefore, some studies are included in the analysis of both follow-up subgroups and one study compared IPS with both an active and passive control group. Therefore, the total amount of studies and sample sizes analysed in each comparison is sometimes lower than the sum of studies analysed in both follow-up subgroups.

b Summary statistics for each of the three employment outcomes are assessed as follows: Employment rate: number and percentage of people in competitive employment at the follow-up assessment; Job duration: percentage of time within the study period that participants are employed; Wages: monthly salary in euros during the study period.

c *d* > 0 and OR > 1 indicates outcomes are beneficial for IPS compared to the control group; *d* < 0 and OR < 1 indicates outcomes are beneficial for the control group compared to IPS.

d Magnitude of effect (Chinn, 2000): Not clinically relevant [N]: *d* > −0.2 – <0.2; OR > 0.67 – <1.5; Small effect [S]: *d* ⩽ −0.20 and >−0.50 – ⩾0.20 and <0.50; OR ⩽ 0.67 and >0.29 – ⩾1.5 and <3.5; Medium effect [M]: *d* ⩽ −0.50 and >−0.80 – ⩾0.50 and <0.80; OR ⩽ 0.29 and >0.20 – ⩾3.5 and <5; Large effect [L]: *d* < −0.80 – >0.80; OR < 0.20 – >5.

Twenty-three studies reported job duration outcomes. Results indicated that IPS participants were longer employed than those in the control group during follow-up, showing small effect sizes (*d* = 0.41 [0.30–0.52], *p* < 0.01). Outcomes were moderately heterogeneous (*I^2^* = 77% [69–83%]; *p* < 0.01). The overall effect sizes of job duration were not influenced by the type of control group, follow-up duration or region.

Fifteen studies reported outcomes of wages. Results indicated that IPS participants earned more wages during the study period than those in the control group, though effect sizes were small (*d* = 0.31 [0.19–0.44], *p* < 0.01). Outcomes were moderately heterogeneous (*I^2^* = 65% [51–76%]; *p* < 0.01). The overall effect sizes of wages were not influenced by the type of control group, follow-up duration or region.

### Moderating effects on overall outcomes

**Fig. 3.**
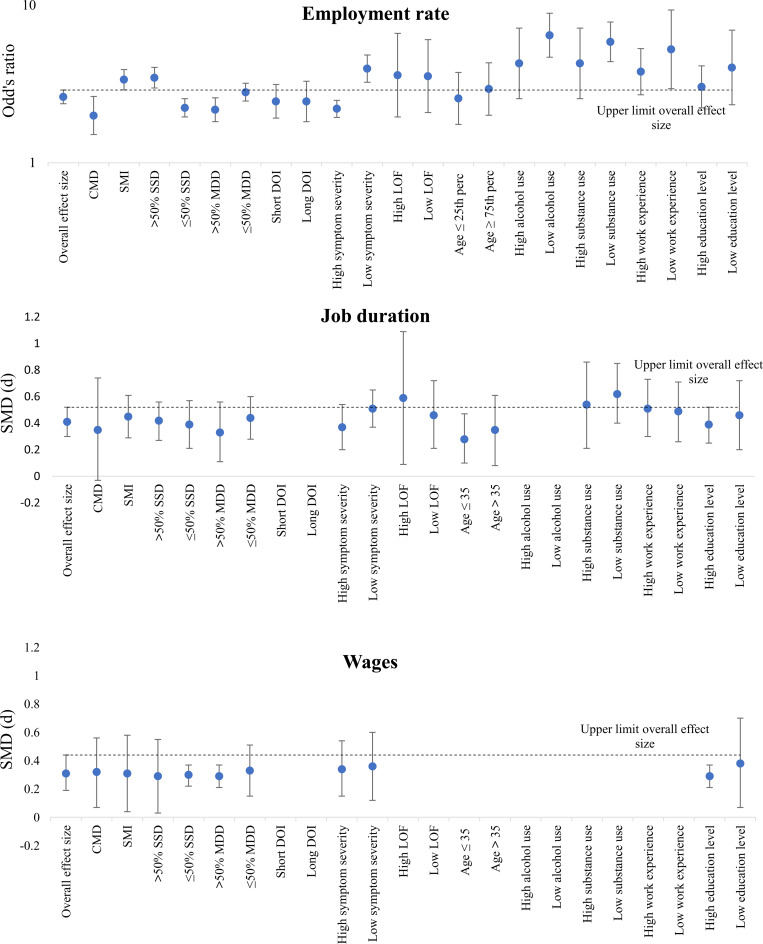
Overview effect sizes of outcomes for each moderator.

Sensitivity analysis outcomes were reported in Table 4 and Fig. 3. We excluded some moderators in the sensitivity analysis of job duration and wages, because these moderators were reported in less than ten studies.

**Table 4.** Sensitivity analysis of moderating effects on the outcomes

Employment rate													
*Research question 1: Effectiveness IPS for different diagnoses*								Effect size of outcome^a^			Heterogeneity		
Moderator^b^	Subgroups	*N* studies^c^	N sample (IPS – control)	*n* (%) at FU^d^				OR^b^	95% CI	*p*	*I*^2^	95% CI	*p*
				IPS		Control							
				*n* (%)	*N*	*n* (%)	*N*						
Severity of psychiatric disorders	CMD	4	445–420	243 (54.6%)	445	161 (38.3%)	420	1.99 [S]	1.51–2.63	<0.01	72%	24–90%	0.01
	SMI	20	1751–1706	795 (45.4%)	1751	408 (23.9%)	1706	3.37 [S]	2.90–3.90	<0.01	76%	68–83%	<0.01
	Subgroup difference		*χ*^2^ = 10.79; df = 1; *p* < 0.01										
Schizophrenia spectrum disorder (SSD)	>50% of study sample SSD	20	1574–1578	763 (48.5%)	1574	396 (25.1%)	1578	3.46 [S]	2.98–4.03	<0.01	75%	66–82%	<0.01
	⩽ 50% of study sample SSD	10	1889–1878	974 (51.6%)	1889	615 (32.8%)	1878	2.23 [S]	1.95–2.55	<0.01	57%	34–72%	0.01
	Subgroup difference		*χ*^2^ = 18.24; df = 1; *p* < 0.01										
Majr depressive disorder (MDD)	>50% of study sample MDD	3	1081–1118	542 (50.1%)	1081	357 (31.9%)	1118	2.17 [S]	1.82–2.58	<0.01	0%	0–95%	0.54
	⩽ 50% of study sample MDD	22	2224–2164	1052 (47.3%)	2224	577 (25.7%)	2164	2.80 [S]	2.46–3.19	<0.01	74%	65–80%	<0.01
	Subgroup difference		*χ*^2^ = 5.36; df = 1; *p* < 0.05										
*Research question 2: Effectiveness IPS for different clinical, functional and personal characteristics*								Effect size of outcome^a^			Heterogeneity		
Moderator	Subgroups	*N* studies^c^	*N* sample (IPS – control)	*n* (%) at FU^d^				OR^b^	95% CI	*p*	*I*^2^	95% CI	*p*
				IPS		Control							
				*n* (%)	*N*	*n* (%)	*N*						
Duration of illness at baseline	Short duration of illness	6	617–579	293 (47.5%)	617	156 (26.9%)	579	2.45 [S]	1.92–3.14	<0.01	69%	37–84%	<0.01
	Long duration of illness	5	465–450	221 (47.5%)	465	130 (28.9%)	450	2.45 [S]	1.82–3.29	<0.01	34%	0–60%	0.19
	Subgroup difference		*χ*^2^ = 0.00; df = 1; *p* = 0.99										
Baseline severity of symptoms	High severity of symptoms	11	2105–2099	1055 (50.1%)	2105	672 (32.0%)	2099	2.20 [S]	1.94–2.49	<0.01	70%	54–81%	<0.01
	Low severity of symptoms	12	1035–1046	477 (46.1%)	1035	223 (21.3%)	1046	3.95 [M]	3.24–4.82	<0.01	59%	40–72%	<0.01
	Subgroup difference		*χ*^2^ = 23.99; df = 1; *p* < 0.01										
Baseline Level of Functioning (LOF)	High baseline LOF	7	540–487	274 (50.7%)	540	144 (29.6%)	487	3.59 [M]	1.95–6.61	<0.01	77%	57–88%	<0.01
	Low baseline LOF	7	846–846	428 (50.6%)	846	247 (29.2%)	846	3.54 [M]	2.08–6.04	<0.01	84%	70–91%	<0.01
	Subgroup difference		*χ*^2^ = 0.00; df = 1; *p* = 0.97										
Age at baseline	⩽ 25th percentile	8	631–628	311 (49.3%)	631	202 (32.2%)	628	2.56 [S]	1.75–3.74	<0.01	56%	28–73%	<0.01
	⩾ 75th percentile	8	1699–1728	864 (50.9%)	1699	539 (31.2%)	1728	2.94 [S]	2.00–4.31	<0.01	74%	54–85%	<0.01
	Subgroup difference		*χ*^2^ = 0.24; df = 1; *p* = 0.62										
Comorbid alcohol use at baseline	High alcohol use	6	501–500	308 (61.5%)	501	183 (36.6%)	500	3.51 [M]	1.93–6.39	<0.01	72%	43–86%	<0.01
	Low alcohol use	6	398–405	243 (61.1%)	398	87 (21.5%)	405	6.44 [L]	4.67–8.88	<0.01	14%	0–29%	0.32
	Subgroup difference		*χ*^2^ = 3.05; df = 1; *p* = 0.08										
Comorbid substance use at baseline	High substance use	8	379–365	187 (49.3%)	379	82 (22.5%)	365	4.27 [M]	2.55–7.15	<0.01	47%	19–66%	0.06
	Low substance use	7	494–511	328 (66.4%)	494	132 (25.8%)	511	5.84 [L]	4.38–7.80	<0.01	26%	1–45%	0.23
	Subgroup difference		*χ*^2^ = 1.08; df = 1; *p* = 0.30										
Work experience at baseline	High work experience	8	596–585	282 (49.6%)	596	132 (22.6%)	585	3.78 [M]	2.70–5.30	<0.01	34%	8–52%	0.16
	Low work experience	8	752–750	374 (49.7%)	752	160 (21.3%)	750	5.24 [L]	2.95–9.30	<0.01	83%	69–90%	<0.01
	Subgroup difference		*χ*^2^ = 0.93; df = 1; *p* = 0.34										
Education level: tertiary education	High proportion tertiary education	10	1795–1826	979 (54.5%)	1795	603 (33.0%)	1826	3.03 [S]	2.23–4.12	<0.01	64%	44–77%	<0.01
	Low proportion tertiary education	10	890–856	411 (46.2%)	890	243 (28.4%)	856	4.01 [M]	2.33–6.93	<0.01	83%	73–90%	<0.01
	Subgroup difference		*χ*^2^ = 0.77; df = 1; *p* = 0.38										
Job duration													
*Research question 1: Effectiveness IPS for different diagnoses*								Effect size of outcome^a^			Heterogeneity		
Moderator^b^	Subgroups	*N* studies^c^	*N* sample (IPS – control)	M (s.d.) at FU^d^				*d*^b^	95% CI	*p*	*I*^2^	95% CI	*p*
				IPS		Control							
				M (s.d.)	*N*	M (s.d.)	*N*						
Severity of psychiatric disorders	CMD	3	475–477	30.2 (10.0)	475	17.4 (5.4)	477	0.35 [S]	−0.03 to 0.74	0.07	90%	69–97%	<0.01
	SMI	12	1026–1020	22.1 (12.1)	1026	10.0 (10.9)	1020	0.45 [S]	0.29–0.61	<0.01	78%	66–86%	<0.01
	Subgroup difference		*χ*^2^ = 0.19; df = 1; *p* = 0.66										
Schizophrenia spectrum disorder (SSD)	>50% of study sample SSD	13	1070–1078	21.4 (11.8)	1070	10.2 (10.5)	1078	0.42 [S]	0.27–0.56	<0.01	77%	65–85%	<0.01
	⩽ 50% of study sample SSD	9	1769–1792	30.7 (10.8)	1769	18.0 (12.0)	1792	0.39 [S]	0.21–0.57	<0.01	80%	66–88%	<0.01
	Subgroup difference		*χ*^2^ = 0.04; df = 1; *p* = 0.84										
Major depressive disorder (MDD)	>50% of study sample MDD	3	1199–1240	22.5 (6.6)	1199	13.3 (11.3)	1240	0.33 [S]	0.11–0.56	<0.01	80%	26–95%	<0.01
	⩽ 50% of study sample MDD	16	1453–1431	28.0 (12.6)	1453	14.9 (12.4)	1431	0.44 [S]	0.28–0.60	<0.01	81%	72–87%	<0.01
	Subgroup difference		*χ*^2^ = 0.55; df = 1; *p* = 0.46										
*Research question 2: Effectiveness IPS for different clinical, functional and personal characteristics*								Effect size of outcome^a^			Heterogeneity		
Moderator	Subgroups	*N* studies^c^	*N* sample (IPS – control)	M (s.d.) at FU^d^				*d*^b^	95% CI	*p*	*I*^2^	95% CI	*p*
				IPS		Control							
				M (s.d.)	*N*	M (s.d.)	*N*						
Baseline severity of symptoms	High severity of symptoms	9	1943–1990	21.3 (10.7)	1943	10.6 (6.9)	1990	0.37 [S]	0.20–0.54	<0.01	84%	73–91%	<0.01
	Low severity of symptoms	9	670–665	27.5 (12.3)	670	12.2 (12.2)	665	0.51 [M]	0.37–0.65	<0.01	59%	35–74%	0.01
	Subgroup difference		*χ*^2^ = 1.69; df = 1; *p* = 0.19										
Baseline Level of Functioning (LOF)	High baseline LOF	3	163–162	25.0 (10.4)	163	8.1 (5.6)	162	0.59 [M]	0.09–1.09	0.02	83%	39–96%	<0.01
	Low baseline LOF	7	911–905	26.3 (11.9)	911	11.7 (7.2)	905	0.46 [S]	0.21–0.72	<0.01	88%	78–93%	<0.01
	Subgroup difference		*χ*^2^ = 0.19; df = 1; *p* = 0.66										
Age at baseline	⩽ 25th percentile	6	510–506	27.7 (16.4)	510	16.5 (12.8)	506	0.28 [S]	0.10–0.47	<0.01	52%	13–73%	0.07
	⩾ 75th percentile	5	1512–1552	23.5 (7.5)	1512	13.4 (9.7)	1552	0.35 [S]	0.08–0.61	<0.05	85%	66–93%	<0.01
	Subgroup difference		*χ*^2^ = 0.14; df = 1; *p* = 0.71										
Comorbid substance use at baseline	High substance use	4	115–112	33.6 (16.2)	115	18.7 (16.6)	112	0.54 [M]	0.21–0.86	<0.01	43%	0–73%	0.15
	Low substance use	6	428–425	26.5 (9.6)	428	8.3 (5.7)	425	0.62 [M]	0.40–0.85	<0.01	74%	48–87%	<0.01
	Subgroup difference		*χ*^2^ = 0.19; df = 1; *p* = 0.66										
Work experience at baseline	High work experience	6	382–375	27.3 (15.0)	382	13.7 (14.5)	375	0.51 [M]	0.30–0.73	<0.01	67%	34–83%	0.01
	Low work experience	7	639–643	19.6 (8.9)	639	7.0 (5.4)	643	0.49 [S]	0.26–0.71	<0.01	83%	67–91%	<0.01
	Subgroup difference		*χ*^2^ = 0.03; df = 1; *p* = 0.87										
Education level: tertiary education	High proportion tertiary education	8	1674–1707	32.1 (12.9)	1674	19.8 (14.3)	1707	0.39 [S]	0.25–0.52	<0.01	61%	35–77%	0.01
	Low proportion tertiary education	7	640–630	22.2 (6.5)	640	9.6 (9.7)	630	0.46 [S]	0.20–0.72	<0.01	83%	68–91%	<0.01
	Subgroup difference		*χ*^2^ = 0.24; df = 1; *p* = 0.63										
Wages													
*Research question 1: Effectiveness IPS for different diagnoses*								Effect size of outcome^a^			Heterogeneity		
Moderator^b^	Subgroups	*N* studies^c^	*N* sample (IPS – control)	M (s.d.) at FU^d^				*d*^b^	95% CI	*p*	*I*^2^	95% CI	p
				IPS		Control							
				M (s.d.)	*N*	M (s.d.)	*N*						
Severity of psychiatric disorders	CMD	3	357–354	797.2 (214.5)	357	553.9 (388.4)	354	0.32 [S]	0.07–0.56	0.01	41%	0–80%	0.18
	SMI	7	666–719	141.7 (146.3)	666	94.4 (129.8)	719	0.31 [S]	0.04–0.58	0.03	83%	69–91%	<0.01
	Subgroup difference		*χ*^2^ = 0.00; df = 1; *p* = 0.96										
Schizophrenia spectrum disorder (SSD)	>50% of study sample SSD	7	639–698	123.5 (152.6)	639	88.9 (132.8)	698	0.29 [S]	0.03–0.55	0.03	82%	67–91%	<0.05
	⩽ 50% of study sample SSD	7	1491–1523	686.6 (309.0)	1491	479.1 (334.1)	1523	0.30 [S]	0.22–0.37	<0.01	2%	0–4%	0.41
	Subgroup difference		*χ*^2^ = 0.00; df = 1; *p* = 0.96										
Major depressive disorder (MDD)	>50% of study sample MDD	2	1048–1092	737.4 (433.8)	1048	619.2 (480.1)	1092	0.29 [S]	0.21–0.37	<0.01	0%	NA	0.96
	⩽ 50% of study sample MDD	11	1007–1055	387.5 (350.3)	1007	254.1 (274.7)	1055	0.33 [S]	0.15–0.51	<0.01	75%	61–84%	<0.01
	Subgroup difference		*χ*^2^ = 0.15; df = 1; *p* = 0.70										
*Research question 2: Effectiveness IPS for different clinical, functional and personal characteristics*								Effect size of outcome^a^			Heterogeneity		
Moderator	Subgroups	*N* studies^c^	*N* sample (IPS – control)	M (s.d.) at FU^d^				*d*^b^	95% CI	*p*	*I*^2^	95% CI	*p*
				IPS		Control							
				M (s.d.)	*N*	M (s.d.)	*N*						
Baseline severity of symptoms	High severity of symptoms	5	1628–1670	412.6 (270.6)	1628	233.9 (186.9)	1670	0.34 [S]	0.15–0.54	<0.01	81%	56–92%	<0.01
	Low severity of symptoms	5	288–284	335.3 (358.7)	288	199.0 (238.9)	284	0.36 [S]	0.12–0.60	<0.01	61%	16–82%	0.04
	Subgroup difference		*χ*^2^ = 0.01; df = 1; *p* = 0.93										
Education level: tertiary education	High proportion tertiary education	7	1520–1551	564.7 (369.9)	1520	407.8 (259.3)	1551	0.29 [S]	0.21–0.37	<0.01	4%	0–9%	0.40
	Low proportion tertiary education	4	403–467	241.6 (549.9)	403	144.8 (533.3)	467	0.38 [S]	0.07–0.70	0.02	83%	54–94%	<0.01
	Subgroup difference		*χ*^2^ = 0.32; df = 1; *p* = 0.57										

a *d* > 0 and OR > 1 indicates outcomes are beneficial for IPS compared to the control group; *d* < 0 and OR < 1 indicates outcomes are beneficial for the control group compared to IPS.

^b^ Underlined moderators were significant moderators of outcome.

c Magnitude of effect: Not clinically relevant [N]: *d* > −0.2 – <0.2; OR > 0.67 – <1.5; Small effect [S]: *d* ⩽ −0.20 and >−0.50 – ⩾0.20 and <0.50; OR ⩽ 0.67 and >0.29 – ⩾1.5 and <3.5; Medium effect [M]: *d* ⩽ −0.50 and >−0.80 – ⩾0.50 and <0.80; OR ⩽ 0.29 and >0.20 – ⩾3.5 and <5; Large effect [L]: *d* < −0.80 – >0.80; OR < 0.20 – >5.

d Summary statistics for each of the three employment outcomes are assessed as follows: Employment rate: number and percentage of people in competitive employment at the follow-up assessment; Job duration: percentage of time within the study period employed that participants are employed; Wages: monthly salary in euros during the study period.

We found significant favourable employment rate outcomes in the IPS group compared with the control group in all subgroups. However, IPS showed more favourable outcomes in studies targeting participants with SMI than studies targeting CMD (*χ*^2^ = 10.79; df = 1; *p* < 0.01). These differences between both subgroups were specifically explained by differences in employment rates in the control group (i.e., 38.3% in the CMD subgroup versus 23.9% in the SMI subgroup; *χ*^2^ = 28.84; df = 1; *p* < 0.01). We also found more favourable outcomes for IPS in subgroups with a majority diagnosed with SSD (*χ*^2^ = 18.24; df = 1; *p* < 0.01), as well as in subgroups with a minority diagnosed with MDD (*χ*^2^ = 5.36; df = 1; *p* < 0.05), and in subgroups with a lower baseline level of symptoms (*χ*^2^ = 20.48; df = 1; *p* < 0.01).

Figure 3 shows all subgroup outcomes. Subgroups with SMI, the majority diagnosed with SSD, low symptom severity, and low comorbid alcohol and substance use problems at baseline had a positive influence on the relative effectiveness of IPS.

None of the potential moderators included in the sensitivity analysis had significant effects on either job duration and wages.

As the type of control group and the region in which the study is executed significantly influenced employment rate outcomes, above-mentioned moderating effects might be explained by an overrepresentation of a specific moderator in one of the subgroups based on the region or type of control group. However, chi-square analyses did not find any indications of overrepresentation in any of these subgroups. We could therefore not explain any moderating effects by regional differences or type of control group.

### Assessment of outliers and publication bias

We found two negative outliers and six positive outliers for employment rate, three negative outliers and three positive outliers for job duration and one negative and one positive outlier for wages. Removing these outliers did not positively or negatively influence the study outcomes.

The funnel plots are presented in online Supplementary materials 3. For all outcomes (employment rate, job duration and wages) we found no indications of publication bias.

## Discussion

This meta-analysis investigated the relative effectiveness of IPS for different subgroups based on diagnostic, clinical, functional and personal characteristics. Overall, we found that IPS is effective in improving employment outcomes regardless of sample characteristics. However, we did find that IPS was relatively less effective in supporting service users into competitive employment in European studies and in studies comparing IPS to a passive control group. Furthermore, we found that IPS was relatively more effective for people with SMIs, compared with CMD. We also found more favourable outcomes of IPS in subgroups in which the majority was diagnosed with a schizophrenia spectrum disorder (SSD), in which the minority was diagnosed with major depressive disorder (MDD), subgroups with a low baseline symptom severity, and subgroups with a low baseline level of substance and alcohol use problems. These subgroup effects could not be explained by an overrepresentation of non-European studies or an active control group within any subgroup. Despite the fact that we found overall effectiveness of IPS for all subgroups, the issue remains that in many studies the majority of service users that received IPS remain unemployed. This highlights the need for continuous refinement of the IPS model.

The fact that IPS was less effective on employment rate outcomes in European studies is most probably explained by the relatively extensive welfare systems with a disability benefit structure in most European countries. The risk of losing steady income from disability benefits after finding competitive employment (i.e., the ‘benefits trap’) might discourage service users from seeking employment (Burns and Cathy, 2008; Metcalfe *et al*., 2018a).

We also found that IPS was relatively more effective on employment rate outcomes when compared with an active control group than when compared with a passive control group. We found a slightly *larger* employment rate in the IPS group (i.e., 50.1% vs 48.3%) but a slightly *smaller* employment rate in the control group (i.e., 26.7% vs. 29.4%) when IPS was compared with an active control group. However, differences in both IPS and control groups were negligible and therefore we could not give any clinical meaningful explanations for the differences between both types of the control groups.

Our findings that IPS is relatively more effective for people with SMI and SSD and relatively less effective for people with MDD and CMD are in line with previous research (Hellström *et al*., 2021). The main explanation for differences in the effectiveness of IPS between CMD and SMI subgroups is that employment rate outcomes in the control group were larger in the CMD subgroup, whereas the outcomes were equal in the IPS group. Previous research found even more favourable employment outcomes in the control group for people with mood disorders or less severe thought disorders (Campbell *et al*., 2010; Jonsdottir and Waghorn, 2015). This may indicate that people with CMD also benefit from other vocational rehabilitation interventions. Nevertheless, our meta-analysis indicates that IPS leads to more favourable employment outcomes for people with CMD compared to any control group, and these indications of effectiveness were also found in another recently published meta-analysis (Probyn *et al*., 2021).

Another possible explanation for the differences between CMD and SMI subgroups is the fact that IPS is originally developed for people with SMI who are generally supported by professionals working in integrated treatment teams, whereas service users with CMD are often supported in different healthcare settings. Previous research indicated that the level of organisational characteristics, such as the type of clinical practice, service intensity and quality of mental health treatment could be an important prerequisite for successful implementation (Lockett *et al*., 2018). This explanation is supported by two studies that conducted IPS for service users with CMD (Hellström *et al*., 2017; Poremski *et al*., 2017) in another healthcare setting for this group. These differences can also partially be explained by the fidelity scores. Only fifty per cent of all studies that evaluated the effectiveness of IPS for people with CMD reached a fair fidelity. In contrast, 89% of the studies that evaluated the effectiveness of IPS for people with SMI achieved good or excellent fidelity. Given the fact that better fidelity scores lead to better outcomes in IPS (Bond *et al*., 2012; Kim *et al*., 2015; Lockett *et al*., 2016; De Winter *et al*., 2020), this might be an important explanation for the differences in outcomes between SMI and CMD subgroups. In addition to fidelity, other important factors, such as the quality of healthcare services and the intensity of employment support might also be relevant topics for further investigation. Therefore, poorer outcomes for people with CMD compared to people SMI might partially be explained by specific challenges in the implementation of IPS in a different healthcare setting, which underlines our recommendation to adapt implementation for specific subgroups.

We also found more favourable indications of the effectiveness of IPS for people with lower symptom severity and lower comorbid substance and alcohol use problems at the start of IPS. This is in line with previous studies which also indicated that lower symptom severity increased the odds of being employed for people who received any type of vocational rehabilitation (Michon *et al*., 2005; Campbell *et al*., 2010; Nygren *et al*., 2013). This might be explained by the fact that a lower symptom severity frees up more time for and focus on the adequate job support, because less focus on symptom stabilisation and intensive treatment programmes is needed.

The positive influence of low symptom severity on the effectiveness of IPS may contradict the superior outcomes of IPS for people with SMI compared with CMD, as SMIs are generally associated with higher symptom severity. However, four out of the five studies (80%) that reported outcomes of IPS for people with CMD had a high symptom severity at baseline. Therefore, symptom severity and the severity of illness are not interrelated in this meta-analysis.

This meta-analysis had several limitations. First, all our findings were analysed on a study level and subgroups were based on aggregated scores or percentages of the whole study sample. This analysis provides an overarching overview of the influence of specific service users' characteristics on the effectiveness of IPS, but does not reflect on the specific variability of individual client level characteristics or outcomes. However, despite this limitation, this meta-analysis gives valuable insights toward better understanding of making effective adaptations in the implementation of IPS in real-world settings. Analysis of outcomes on a study level inevitably leads to heterogeneity of outcomes because the context and setting in which the studies are executed differ (Ioannidis, 2008). Furthermore, this meta-analysis only focused on the effectiveness of IPS as a stand-alone intervention within a mental health population, in order to achieve a relatively homogeneous sample of studies. As a consequence, we did not include a number of relevant studies that exclusively investigating IPS with an add-on intervention (e.g., McGurk *et al*., 2015; Tsang *et al*., 2016) or studies focused on populations with a high risk of developing mental disorders (e.g., Sveinsdottir *et al*., 2019). This also partially explained the lack of available study data to investigate the influence of other relevant moderators (such as cognitive functioning). Second, some of our sensitivity analyses, in which we investigated moderating effects, were based on a relatively low number of studies. This might have limited the generalisability of outcomes for subgroups based on a small number of studies. Another limitation is that 12 (37.5%) of our included studies are conducted more than 10 years ago. During that time IPS was executed in a societal setting, that was applicable at that time, with most probably other welfare policies or treatment practices than implemented nowadays. Another potential limitation is the fact that the broad variety of studies might influence the interpretation and representativeness of some moderating effects. Our included studies investigated target groups with different diagnoses and clinical characteristics and were therefore in some cases using different assessment instruments. This was specifically the case in the moderating effects of the severity of symptoms and level of functioning. We tried to solve this issue by using normative data based on representative target groups that matches with each included study. However, this inevitably leads to heterogeneity in the outcomes. Interpretation of the findings on the influence of symptom severity and level of functioning on employment should therefore be handled with caution. Finally, we have executed a relatively high number of sensitivity analyses based on a relatively low number of studies. This increases the chance of false-positive outcomes and alpha inflation (Wang *et al*., 2017). We should therefore consider the results of this meta-analysis as exploratory and the findings suggesting potentially valuable trends for improving IPS for different target groups.

Overall this meta-analysis has shown that IPS is implemented for a wide variety of service users: IPS is effective for different subgroups, regardless of distinct diagnostic, clinical, functional and personal characteristics. However, future research should focus on the implementation of IPS for people with CMD and higher symptom severity. It is important to investigate whether, and if so, how to make more effective adaptations in the implementation of IPS to better meet the vocational needs of these groups.

## Supplementary material

For supplementary material accompanying this paper visit https://doi.org/10.1017/S2045796022000300. click here to view supplementary material

## Availability of data and materials

The majority of relevant data and materials are presented in the tables and online Supplementary materials, as well as partially available in the review protocol that is submitted in PROSPERO (CRD42020220080). All other raw data is not available online. Further questions and requests about the availability of the data could be sent to the corresponding authors (Lars de Winter; Lwinter@kcphrenos.nl).
