# Supplementary material for: Who benefits from individual placement and support? A meta-analysis
Source: Epidemiol Psychiatr Sci. 2022 Jul 11;31:e50. doi: 10.1017/S2045796022000300 (PMC9281491; doi:10.1017/S2045796022000300)
Supplement: Supplementary file 1 [file S2045796022000300sup001.docx]

**Supplementary materials 1. Search history**

**PsycInfo**

Loading...

Bovenkant formulier

| **#** | **Query** | **Limiters/Expanders** | **Last Run Via** | **Results** | **Action** |
| --- | --- | --- | --- | --- | --- |
| S4 | S1 AND S2 AND S3 | Search modes - Boolean/Phrase | Interface - EBSCOhost Research Databases  Search Screen - Advanced Search  Database - PsycINFO | 717 |  |
| S3 | ( ((DE "Occupations" OR DE "Employment Status" OR DE "Job Characteristics" OR DE "Job Search" OR DE "Nontraditional Careers" OR DE "Occupational Choice" OR DE "Occupational Mobility" OR DE "Occupational Tenure" OR DE "Employee Engagement" OR DE "Career Development" OR DE "Work Scheduling" OR DE "Work (Attitudes Toward)" OR DE "Work Week Length" OR DE "Work Related Illnesses" OR DE "Work Load" OR DE "School to Work Transition" OR DE "Work-Life Balance" OR DE "Quality of Work Life" OR DE "Working Conditions" OR DE "Job Enrichment" OR DE "Noise Levels (Work Areas)" OR DE "Occupational Safety" OR DE "Telecommuting" OR DE "Work Rest Cycles" OR DE "Work Week Length" OR DE "Workday Shifts" OR DE "Working Space" OR DE "Reemployment" OR DE "Job Satisfaction") OR (DE "Job Characteristics" OR DE "Job Security" OR DE "Work Load" OR DE "Work Scheduling" OR DE "On the Job Training" OR DE "Job Search" OR DE "Job Satisfaction" OR DE "Job Performance" OR DE "Job Knowledge" OR DE "Job Involvement" OR DE "Personnel Training" OR DE "Apprenticeship" OR DE "Inservice Training" OR DE "Management Training" OR DE "On the Job Training" OR DE "Occupational Stress" OR DE "Occupational Status" OR DE "Occupational Choice" OR DE "Career Change" OR DE "Working Conditions" OR DE "Job Enrichment" OR DE "Noise Levels (Work Areas)" OR DE "Occupational Safety" OR DE "Telecommuting" OR DE "Work Rest Cycles" OR DE "Work Week Length" OR DE "Workday Shifts" OR DE "Working Space" OR DE "Work (Attitudes Toward)" OR DE "Employee Engagement" OR DE "Employability")) OR (DE "Employment Status" OR DE "Employability" OR DE "Employment History" OR DE "Reemployment" OR DE "Retirement" OR DE "Self-Employment" OR DE "Unemployment" OR DE "Employment History" OR DE "Employment Discrimination" OR DE "Job Security") ) OR TX competitive employment OR TX competitive job OR TX paid employment OR TX participation OR TX job performance | Limiters - Published Date: 19900101-20191231  Search modes - Boolean/Phrase | Interface - EBSCOhost Research Databases  Search Screen - Advanced Search  Database - PsycINFO | 212,599 |  |
| S2 | ( (DE "Rehabilitation" OR DE "Cognitive Rehabilitation" OR DE "Criminal Rehabilitation" OR DE "Neuropsychological Rehabilitation" OR DE "Neurorehabilitation" OR DE "Occupational Therapy" OR DE "Physical Therapy" OR DE "Psychosocial Rehabilitation" OR DE "Rehabilitation Centers" OR DE "Telerehabilitation" OR DE "Vocational Rehabilitation" OR DE "Supported Employment" OR DE "Vocational Evaluation" OR DE "Work Adjustment Training" OR DE "Psychosocial Rehabilitation" OR DE "Psychosocial Readjustment" OR DE "Therapeutic Social Clubs" OR DE "Vocational Rehabilitation") OR (MM "On the Job Training" OR MM "Job Search" OR MM "Job Involvement" OR MM "Reemployment" OR DE "Personnel Training" OR DE "Apprenticeship" OR DE "Inservice Training" OR DE "Management Training" OR DE "On the Job Training") ) OR TX ( individual placement and support ) OR TX ips OR TX supported employment OR TX job coaching OR TX work rehabilitation | Limiters - Published Date: 19900101-20191231  Search modes - Boolean/Phrase | Interface - EBSCOhost Research Databases  Search Screen - Advanced Search  Database - PsycINFO | 53,475 |  |
| S1 | ( ((((((((DE "Major Depression" OR DE "Anaclitic Depression" OR DE "Dysthymic Disorder" OR DE "Endogenous Depression" OR DE "Late Life Depression" OR DE "Postpartum Depression" OR DE "Reactive Depression" OR DE "Recurrent Depression" OR DE "Treatment Resistant Depression") AND (DE "Anxiety" OR DE "Anxiety Sensitivity" OR DE "Computer Anxiety" OR DE "Health Anxiety" OR DE "Mathematics Anxiety" OR DE "Performance Anxiety" OR DE "Social Anxiety" OR DE "Speech Anxiety" OR DE "Test Anxiety" OR DE "Anxiety Disorders" OR DE "Castration Anxiety" OR DE "Death Anxiety" OR DE "Generalized Anxiety Disorder" OR DE "Obsessive Compulsive Disorder" OR DE "Panic Attack" OR DE "Panic Disorder" OR DE "Phobias" OR DE "Separation Anxiety Disorder" OR DE "Trichotillomania")) OR (DE "Psychosis" OR DE "Acute Psychosis" OR DE "Affective Psychosis" OR DE "Alcoholic Psychosis" OR DE "Capgras Syndrome" OR DE "Childhood Psychosis" OR DE "Chronic Psychosis" OR DE "Experimental Psychosis" OR DE "Hallucinosis" OR DE "Paranoia (Psychosis)" OR DE "Postpartum Psychosis" OR DE "Reactive Psychosis" OR DE "Schizophrenia" OR DE "Senile Psychosis" OR DE "Toxic Psychoses")) AND (DE "Substance Related and Addictive Disorders" OR DE "Nonsubstance Related Addictions" OR DE "Substance Use Disorder" OR DE "Substance Use Disorder" OR DE "Addiction" OR DE "Alcohol Use Disorder" OR DE "Cannabis Use Disorder" OR DE "Drug Abuse" OR DE "Drug Dependency" OR DE "Inhalant Abuse" OR DE "Opioid Use Disorder" OR DE "Tobacco Use Disorder")) OR (DE "Personality Disorders" OR DE "Antisocial Personality Disorder" OR DE "Avoidant Personality Disorder" OR DE "Borderline Personality Disorder" OR DE "Dependent Personality Disorder" OR DE "Histrionic Personality Disorder" OR DE "Narcissistic Personality Disorder" OR DE "Obsessive Compulsive Personality Disorder" OR DE "Paranoid Personality Disorder" OR DE "Passive Aggressive Personality Disorder" OR DE "Sadomasochistic Personality" OR DE "Schizoid Personality Disorder" OR DE "Schizotypal Personality Disorder")) OR (DE "Bipolar Disorder" OR DE "Bipolar I Disorder" OR DE "Bipolar II Disorder" OR DE "Cyclothymic Disorder" OR DE "Mania")) OR (DE "Stress and Trauma Related Disorders" OR DE "Acute Stress Disorder" OR DE "Adjustment Disorders" OR DE "Attachment Disorders" OR DE "Posttraumatic Stress Disorder")) OR (DE "Obsessive Compulsive Disorder" OR DE "Hoarding Disorder" OR DE "Koro")) OR (DE "Somatoform Disorders" OR DE "Body Dysmorphic Disorder" OR DE "Conversion Disorder" OR DE "Factitious Disorders" OR DE "Hypochondriasis" OR DE "Hysteria" OR DE "Neurasthenia" OR DE "Neurodermatitis" OR DE "Somatization Disorder" OR DE "Somatoform Pain Disorder") ) OR TX common mental disorders OR TX common mental health issues OR TX common mental health disorders OR TX severe mental illness OR TX severe mental disorder OR TX serious mental illness | Limiters - Published Date: 19900101-20191231  Search modes - Boolean/Phrase | Interface - EBSCOhost Research Databases  Search Screen - Advanced Search  Database - PsycINFO | 126,763 |  |

Onderkant formulier

**PubMed**

| Search | Query | Items found | Time |
| --- | --- | --- | --- |
| #36 | Search ((((((((((((((("Depression/analysis"[Mesh] OR "Depression/diagnosis"[Mesh] OR "Depression/diagnostic imaging"[Mesh] OR "Depression/epidemiology"[Mesh] OR "Depression/etiology"[Mesh] OR "Depression/history"[Mesh] OR "Depression/pathology"[Mesh] OR "Depression/physiology"[Mesh] OR "Depression/physiopathology"[Mesh] OR "Depression/prevention and control"[Mesh] OR "Depression/psychology"[Mesh] OR "Depression/rehabilitation"[Mesh] OR "Depression/statistics and numerical data"[Mesh])) OR ("Mood Disorders/analysis"[Mesh] OR "Mood Disorders/classification"[Mesh] OR "Mood Disorders/diagnosis"[Mesh] OR "Mood Disorders/epidemiology"[Mesh] OR "Mood Disorders/etiology"[Mesh] OR "Mood Disorders/history"[Mesh] OR "Mood Disorders/mortality"[Mesh] OR "Mood Disorders/pathology"[Mesh] OR "Mood Disorders/physiology"[Mesh] OR "Mood Disorders/physiopathology"[Mesh] OR "Mood Disorders/prevention and control"[Mesh] OR "Mood Disorders/psychology"[Mesh] OR "Mood Disorders/rehabilitation"[Mesh] OR "Mood Disorders/therapy"[Mesh])) OR ("Anxiety Disorders/analysis"[Mesh] OR "Anxiety Disorders/diagnosis"[Mesh] OR "Anxiety Disorders/drug therapy"[Mesh] OR "Anxiety Disorders/epidemiology"[Mesh] OR "Anxiety Disorders/etiology"[Mesh] OR "Anxiety Disorders/history"[Mesh] OR "Anxiety Disorders/mortality"[Mesh] OR "Anxiety Disorders/physiopathology"[Mesh] OR "Anxiety Disorders/prevention and control"[Mesh] OR "Anxiety Disorders/psychology"[Mesh] OR "Anxiety Disorders/rehabilitation"[Mesh] OR "Anxiety Disorders/statistics and numerical data"[Mesh] OR "Anxiety Disorders/therapeutic use"[Mesh] OR "Anxiety Disorders/therapy"[Mesh])) OR ("Bipolar and Related Disorders/analysis"[Mesh] OR "Bipolar and Related Disorders/classification"[Mesh] OR "Bipolar and Related Disorders/diagnosis"[Mesh] OR "Bipolar and Related Disorders/epidemiology"[Mesh] OR "Bipolar and Related Disorders/etiology"[Mesh] OR "Bipolar and Related Disorders/history"[Mesh] OR "Bipolar and Related Disorders/mortality"[Mesh] OR "Bipolar and Related Disorders/pathology"[Mesh] OR "Bipolar and Related Disorders/physiopathology"[Mesh] OR "Bipolar and Related Disorders/prevention and control"[Mesh] OR "Bipolar and Related Disorders/psychology"[Mesh] OR "Bipolar and Related Disorders/rehabilitation"[Mesh] OR "Bipolar and Related Disorders/statistics and numerical data"[Mesh] OR "Bipolar and Related Disorders/therapy"[Mesh])) OR ("Personality Disorders/analysis"[Mesh] OR "Personality Disorders/classification"[Mesh] OR "Personality Disorders/diagnosis"[Mesh] OR "Personality Disorders/epidemiology"[Mesh] OR "Personality Disorders/ethnology"[Mesh] OR "Personality Disorders/etiology"[Mesh] OR "Personality Disorders/mortality"[Mesh] OR "Personality Disorders/pathology"[Mesh] OR "Personality Disorders/physiology"[Mesh] OR "Personality Disorders/physiopathology"[Mesh] OR "Personality Disorders/prevention and control"[Mesh] OR "Personality Disorders/psychology"[Mesh] OR "Personality Disorders/rehabilitation"[Mesh] OR "Personality Disorders/therapy"[Mesh])) OR "Schizophrenia Spectrum and Other Psychotic Disorders/analysis"[Mesh] OR "Schizophrenia Spectrum and Other Psychotic Disorders/classification"[Mesh] OR "Schizophrenia Spectrum and Other Psychotic Disorders/diagnosis"[Mesh] OR schizophrenia spectrum and other psychotic)) OR severe mental illness[Title/Abstract]) OR serious mental illness[Title/Abstract]) OR smi[Title/Abstract]) OR common mental disorder[Title/Abstract]) OR cmd[Title/Abstract])) AND (((((((((((((((( "Employment/economics"[Mesh] OR "Employment/history"[Mesh] OR "Employment/psychology"[Mesh] OR "Employment/statistics and numerical data"[Mesh] )) OR ( "Work/adverse effects"[Mesh] OR "Work/analysis"[Mesh] OR "Work/classification"[Mesh] OR "Work/history"[Mesh] OR "Work/methods"[Mesh] OR "Work/physiology"[Mesh] OR "Work/psychology"[Mesh] OR "Work/statistics and numerical data"[Mesh] OR "Work/therapeutic use"[Mesh] )) OR "Job Satisfaction/statistics and numerical data"[Mesh]) OR ( "Employment, Supported/economics"[Mesh] OR "Employment, Supported/methods"[Mesh] OR "Employment, Supported/organization and administration"[Mesh] OR "Employment, Supported/psychology"[Mesh] OR "Employment, Supported/standards"[Mesh] OR "Employment, Supported/statistics and numerical data"[Mesh] )) OR ( "Return to Work/psychology"[Mesh] OR "Return to Work/statistics and numerical data"[Mesh] OR "Return to Work/trends"[Mesh] )) OR "Work Engagement"[Mesh]) OR ( "Community Participation/history"[Mesh] OR "Community Participation/methods"[Mesh] OR "Community Participation/psychology"[Mesh] OR "Community Participation/standards"[Mesh] OR "Community Participation/statistics and numerical data"[Mesh] OR "Community Participation/trends"[Mesh] )) OR ( "Social Participation/history"[Mesh] OR "Social Participation/psychology"[Mesh] ))) OR competitive employment[Title/Abstract]) OR employment[Title/Abstract]) OR competitive work[Title/Abstract]) OR work[Title/Abstract]) OR competitive job[Title/Abstract]) OR job[Title/Abstract])) AND ((((((((((( "Rehabilitation, Vocational/classification"[Mesh] OR "Rehabilitation, Vocational/education"[Mesh] OR "Rehabilitation, Vocational/epidemiology"[Mesh] OR "Rehabilitation, Vocational/legislation and jurisprudence"[Mesh] OR "Rehabilitation, Vocational/methods"[Mesh] OR "Rehabilitation, Vocational/mortality"[Mesh] OR "Rehabilitation, Vocational/psychology"[Mesh] OR "Rehabilitation, Vocational/statistics and numerical data"[Mesh] )) OR ( "Employment, Supported/classification"[Mesh] OR "Employment, Supported/economics"[Mesh] OR "Employment, Supported/methods"[Mesh] OR "Employment, Supported/psychology"[Mesh] OR "Employment, Supported/standards"[Mesh] OR "Employment, Supported/statistics and numerical data"[Mesh] )) OR ( "Inservice Training/classification"[Mesh] OR "Inservice Training/economics"[Mesh] OR "Inservice Training/history"[Mesh] OR "Inservice Training/organization and administration"[Mesh] OR "Inservice Training/statistics and numerical data"[Mesh] ))) OR individual placement support[Title/Abstract]) OR individual placement[Title/Abstract]) OR IPS[Title/Abstract]) OR job support[Title/Abstract]) OR supported employment[Title/Abstract]) OR SE[Title/Abstract]) Filters: Publication date from 1990/01/01 to 2019/12/31 | 550 | 7:32:06 |
| #35 | Search ((((((((((((((("Depression/analysis"[Mesh] OR "Depression/diagnosis"[Mesh] OR "Depression/diagnostic imaging"[Mesh] OR "Depression/epidemiology"[Mesh] OR "Depression/etiology"[Mesh] OR "Depression/history"[Mesh] OR "Depression/pathology"[Mesh] OR "Depression/physiology"[Mesh] OR "Depression/physiopathology"[Mesh] OR "Depression/prevention and control"[Mesh] OR "Depression/psychology"[Mesh] OR "Depression/rehabilitation"[Mesh] OR "Depression/statistics and numerical data"[Mesh])) OR ("Mood Disorders/analysis"[Mesh] OR "Mood Disorders/classification"[Mesh] OR "Mood Disorders/diagnosis"[Mesh] OR "Mood Disorders/epidemiology"[Mesh] OR "Mood Disorders/etiology"[Mesh] OR "Mood Disorders/history"[Mesh] OR "Mood Disorders/mortality"[Mesh] OR "Mood Disorders/pathology"[Mesh] OR "Mood Disorders/physiology"[Mesh] OR "Mood Disorders/physiopathology"[Mesh] OR "Mood Disorders/prevention and control"[Mesh] OR "Mood Disorders/psychology"[Mesh] OR "Mood Disorders/rehabilitation"[Mesh] OR "Mood Disorders/therapy"[Mesh])) OR ("Anxiety Disorders/analysis"[Mesh] OR "Anxiety Disorders/diagnosis"[Mesh] OR "Anxiety Disorders/drug therapy"[Mesh] OR "Anxiety Disorders/epidemiology"[Mesh] OR "Anxiety Disorders/etiology"[Mesh] OR "Anxiety Disorders/history"[Mesh] OR "Anxiety Disorders/mortality"[Mesh] OR "Anxiety Disorders/physiopathology"[Mesh] OR "Anxiety Disorders/prevention and control"[Mesh] OR "Anxiety Disorders/psychology"[Mesh] OR "Anxiety Disorders/rehabilitation"[Mesh] OR "Anxiety Disorders/statistics and numerical data"[Mesh] OR "Anxiety Disorders/therapeutic use"[Mesh] OR "Anxiety Disorders/therapy"[Mesh])) OR ("Bipolar and Related Disorders/analysis"[Mesh] OR "Bipolar and Related Disorders/classification"[Mesh] OR "Bipolar and Related Disorders/diagnosis"[Mesh] OR "Bipolar and Related Disorders/epidemiology"[Mesh] OR "Bipolar and Related Disorders/etiology"[Mesh] OR "Bipolar and Related Disorders/history"[Mesh] OR "Bipolar and Related Disorders/mortality"[Mesh] OR "Bipolar and Related Disorders/pathology"[Mesh] OR "Bipolar and Related Disorders/physiopathology"[Mesh] OR "Bipolar and Related Disorders/prevention and control"[Mesh] OR "Bipolar and Related Disorders/psychology"[Mesh] OR "Bipolar and Related Disorders/rehabilitation"[Mesh] OR "Bipolar and Related Disorders/statistics and numerical data"[Mesh] OR "Bipolar and Related Disorders/therapy"[Mesh])) OR ("Personality Disorders/analysis"[Mesh] OR "Personality Disorders/classification"[Mesh] OR "Personality Disorders/diagnosis"[Mesh] OR "Personality Disorders/epidemiology"[Mesh] OR "Personality Disorders/ethnology"[Mesh] OR "Personality Disorders/etiology"[Mesh] OR "Personality Disorders/mortality"[Mesh] OR "Personality Disorders/pathology"[Mesh] OR "Personality Disorders/physiology"[Mesh] OR "Personality Disorders/physiopathology"[Mesh] OR "Personality Disorders/prevention and control"[Mesh] OR "Personality Disorders/psychology"[Mesh] OR "Personality Disorders/rehabilitation"[Mesh] OR "Personality Disorders/therapy"[Mesh])) OR "Schizophrenia Spectrum and Other Psychotic Disorders/analysis"[Mesh] OR "Schizophrenia Spectrum and Other Psychotic Disorders/classification"[Mesh] OR "Schizophrenia Spectrum and Other Psychotic Disorders/diagnosis"[Mesh] OR schizophrenia spectrum and other psychotic)) OR severe mental illness[Title/Abstract]) OR serious mental illness[Title/Abstract]) OR smi[Title/Abstract]) OR common mental disorder[Title/Abstract]) OR cmd[Title/Abstract])) AND (((((((((((((((( "Employment/economics"[Mesh] OR "Employment/history"[Mesh] OR "Employment/psychology"[Mesh] OR "Employment/statistics and numerical data"[Mesh] )) OR ( "Work/adverse effects"[Mesh] OR "Work/analysis"[Mesh] OR "Work/classification"[Mesh] OR "Work/history"[Mesh] OR "Work/methods"[Mesh] OR "Work/physiology"[Mesh] OR "Work/psychology"[Mesh] OR "Work/statistics and numerical data"[Mesh] OR "Work/therapeutic use"[Mesh] )) OR "Job Satisfaction/statistics and numerical data"[Mesh]) OR ( "Employment, Supported/economics"[Mesh] OR "Employment, Supported/methods"[Mesh] OR "Employment, Supported/organization and administration"[Mesh] OR "Employment, Supported/psychology"[Mesh] OR "Employment, Supported/standards"[Mesh] OR "Employment, Supported/statistics and numerical data"[Mesh] )) OR ( "Return to Work/psychology"[Mesh] OR "Return to Work/statistics and numerical data"[Mesh] OR "Return to Work/trends"[Mesh] )) OR "Work Engagement"[Mesh]) OR ( "Community Participation/history"[Mesh] OR "Community Participation/methods"[Mesh] OR "Community Participation/psychology"[Mesh] OR "Community Participation/standards"[Mesh] OR "Community Participation/statistics and numerical data"[Mesh] OR "Community Participation/trends"[Mesh] )) OR ( "Social Participation/history"[Mesh] OR "Social Participation/psychology"[Mesh] ))) OR competitive employment[Title/Abstract]) OR employment[Title/Abstract]) OR competitive work[Title/Abstract]) OR work[Title/Abstract]) OR competitive job[Title/Abstract]) OR job[Title/Abstract])) AND ((((((((((( "Rehabilitation, Vocational/classification"[Mesh] OR "Rehabilitation, Vocational/education"[Mesh] OR "Rehabilitation, Vocational/epidemiology"[Mesh] OR "Rehabilitation, Vocational/legislation and jurisprudence"[Mesh] OR "Rehabilitation, Vocational/methods"[Mesh] OR "Rehabilitation, Vocational/mortality"[Mesh] OR "Rehabilitation, Vocational/psychology"[Mesh] OR "Rehabilitation, Vocational/statistics and numerical data"[Mesh] )) OR ( "Employment, Supported/classification"[Mesh] OR "Employment, Supported/economics"[Mesh] OR "Employment, Supported/methods"[Mesh] OR "Employment, Supported/psychology"[Mesh] OR "Employment, Supported/standards"[Mesh] OR "Employment, Supported/statistics and numerical data"[Mesh] )) OR ( "Inservice Training/classification"[Mesh] OR "Inservice Training/economics"[Mesh] OR "Inservice Training/history"[Mesh] OR "Inservice Training/organization and administration"[Mesh] OR "Inservice Training/statistics and numerical data"[Mesh] ))) OR individual placement support[Title/Abstract]) OR individual placement[Title/Abstract]) OR IPS[Title/Abstract]) OR job support[Title/Abstract]) OR supported employment[Title/Abstract]) OR SE[Title/Abstract]) | 566 | 7:31:52 |
| #34 | Search (((((((((( "Rehabilitation, Vocational/classification"[Mesh] OR "Rehabilitation, Vocational/education"[Mesh] OR "Rehabilitation, Vocational/epidemiology"[Mesh] OR "Rehabilitation, Vocational/legislation and jurisprudence"[Mesh] OR "Rehabilitation, Vocational/methods"[Mesh] OR "Rehabilitation, Vocational/mortality"[Mesh] OR "Rehabilitation, Vocational/psychology"[Mesh] OR "Rehabilitation, Vocational/statistics and numerical data"[Mesh] )) OR ( "Employment, Supported/classification"[Mesh] OR "Employment, Supported/economics"[Mesh] OR "Employment, Supported/methods"[Mesh] OR "Employment, Supported/psychology"[Mesh] OR "Employment, Supported/standards"[Mesh] OR "Employment, Supported/statistics and numerical data"[Mesh] )) OR ( "Inservice Training/classification"[Mesh] OR "Inservice Training/economics"[Mesh] OR "Inservice Training/history"[Mesh] OR "Inservice Training/organization and administration"[Mesh] OR "Inservice Training/statistics and numerical data"[Mesh] ))) OR individual placement support[Title/Abstract]) OR individual placement[Title/Abstract]) OR IPS[Title/Abstract]) OR job support[Title/Abstract]) OR supported employment[Title/Abstract]) OR SE[Title/Abstract] | 127378 | 7:30:40 |
| #28 | Search ((((((((((((((( "Employment/economics"[Mesh] OR "Employment/history"[Mesh] OR "Employment/psychology"[Mesh] OR "Employment/statistics and numerical data"[Mesh] )) OR ( "Work/adverse effects"[Mesh] OR "Work/analysis"[Mesh] OR "Work/classification"[Mesh] OR "Work/history"[Mesh] OR "Work/methods"[Mesh] OR "Work/physiology"[Mesh] OR "Work/psychology"[Mesh] OR "Work/statistics and numerical data"[Mesh] OR "Work/therapeutic use"[Mesh] )) OR "Job Satisfaction/statistics and numerical data"[Mesh]) OR ( "Employment, Supported/economics"[Mesh] OR "Employment, Supported/methods"[Mesh] OR "Employment, Supported/organization and administration"[Mesh] OR "Employment, Supported/psychology"[Mesh] OR "Employment, Supported/standards"[Mesh] OR "Employment, Supported/statistics and numerical data"[Mesh] )) OR ( "Return to Work/psychology"[Mesh] OR "Return to Work/statistics and numerical data"[Mesh] OR "Return to Work/trends"[Mesh] )) OR "Work Engagement"[Mesh]) OR ( "Community Participation/history"[Mesh] OR "Community Participation/methods"[Mesh] OR "Community Participation/psychology"[Mesh] OR "Community Participation/standards"[Mesh] OR "Community Participation/statistics and numerical data"[Mesh] OR "Community Participation/trends"[Mesh] )) OR ( "Social Participation/history"[Mesh] OR "Social Participation/psychology"[Mesh] ))) OR competitive employment[Title/Abstract]) OR employment[Title/Abstract]) OR competitive work[Title/Abstract]) OR work[Title/Abstract]) OR competitive job[Title/Abstract]) OR job[Title/Abstract] | 996265 | 7:18:36 |
| #16 | Search (((((((((((("Depression/analysis"[Mesh] OR "Depression/diagnosis"[Mesh] OR "Depression/diagnostic imaging"[Mesh] OR "Depression/epidemiology"[Mesh] OR "Depression/etiology"[Mesh] OR "Depression/history"[Mesh] OR "Depression/pathology"[Mesh] OR "Depression/physiology"[Mesh] OR "Depression/physiopathology"[Mesh] OR "Depression/prevention and control"[Mesh] OR "Depression/psychology"[Mesh] OR "Depression/rehabilitation"[Mesh] OR "Depression/statistics and numerical data"[Mesh])) OR ("Mood Disorders/analysis"[Mesh] OR "Mood Disorders/classification"[Mesh] OR "Mood Disorders/diagnosis"[Mesh] OR "Mood Disorders/epidemiology"[Mesh] OR "Mood Disorders/etiology"[Mesh] OR "Mood Disorders/history"[Mesh] OR "Mood Disorders/mortality"[Mesh] OR "Mood Disorders/pathology"[Mesh] OR "Mood Disorders/physiology"[Mesh] OR "Mood Disorders/physiopathology"[Mesh] OR "Mood Disorders/prevention and control"[Mesh] OR "Mood Disorders/psychology"[Mesh] OR "Mood Disorders/rehabilitation"[Mesh] OR "Mood Disorders/therapy"[Mesh])) OR ("Anxiety Disorders/analysis"[Mesh] OR "Anxiety Disorders/diagnosis"[Mesh] OR "Anxiety Disorders/drug therapy"[Mesh] OR "Anxiety Disorders/epidemiology"[Mesh] OR "Anxiety Disorders/etiology"[Mesh] OR "Anxiety Disorders/history"[Mesh] OR "Anxiety Disorders/mortality"[Mesh] OR "Anxiety Disorders/physiopathology"[Mesh] OR "Anxiety Disorders/prevention and control"[Mesh] OR "Anxiety Disorders/psychology"[Mesh] OR "Anxiety Disorders/rehabilitation"[Mesh] OR "Anxiety Disorders/statistics and numerical data"[Mesh] OR "Anxiety Disorders/therapeutic use"[Mesh] OR "Anxiety Disorders/therapy"[Mesh])) OR ("Bipolar and Related Disorders/analysis"[Mesh] OR "Bipolar and Related Disorders/classification"[Mesh] OR "Bipolar and Related Disorders/diagnosis"[Mesh] OR "Bipolar and Related Disorders/epidemiology"[Mesh] OR "Bipolar and Related Disorders/etiology"[Mesh] OR "Bipolar and Related Disorders/history"[Mesh] OR "Bipolar and Related Disorders/mortality"[Mesh] OR "Bipolar and Related Disorders/pathology"[Mesh] OR "Bipolar and Related Disorders/physiopathology"[Mesh] OR "Bipolar and Related Disorders/prevention and control"[Mesh] OR "Bipolar and Related Disorders/psychology"[Mesh] OR "Bipolar and Related Disorders/rehabilitation"[Mesh] OR "Bipolar and Related Disorders/statistics and numerical data"[Mesh] OR "Bipolar and Related Disorders/therapy"[Mesh])) OR ("Personality Disorders/analysis"[Mesh] OR "Personality Disorders/classification"[Mesh] OR "Personality Disorders/diagnosis"[Mesh] OR "Personality Disorders/epidemiology"[Mesh] OR "Personality Disorders/ethnology"[Mesh] OR "Personality Disorders/etiology"[Mesh] OR "Personality Disorders/mortality"[Mesh] OR "Personality Disorders/pathology"[Mesh] OR "Personality Disorders/physiology"[Mesh] OR "Personality Disorders/physiopathology"[Mesh] OR "Personality Disorders/prevention and control"[Mesh] OR "Personality Disorders/psychology"[Mesh] OR "Personality Disorders/rehabilitation"[Mesh] OR "Personality Disorders/therapy"[Mesh])) OR "Schizophrenia Spectrum and Other Psychotic Disorders/analysis"[Mesh] OR "Schizophrenia Spectrum and Other Psychotic Disorders/classification"[Mesh] OR "Schizophrenia Spectrum and Other Psychotic Disorders/diagnosis"[Mesh] OR schizophrenia spectrum and other psychotic)) OR severe mental illness[Title/Abstract]) OR serious mental illness[Title/Abstract]) OR smi[Title/Abstract]) OR common mental disorder[Title/Abstract]) OR cmd[Title/Abstract] | 327998 | 7:11:48 |
|  |  |  |  |

**Cochrane**

| **ID** | **Search** | **Hits** |
| --- | --- | --- |
| #1 | MeSH descriptor: [Behavioral Symptoms] explode all trees | 18846 |
| #2 | MeSH descriptor: [Mental Disorders] explode all trees | 65006 |
| #3 | (common mental disorder):ti,ab,kw | 1700 |
| #4 | (severe mental illness):ti,ab,kw | 1669 |
| #5 | (serious mental illness):ti,ab,kw | 981 |
| #6 | (CMD):ti,ab,kw | 164 |
| #7 | (SMI):ti,ab,kw | 569 |
| #8 | #1 OR #2 OR #3 OR #4 OR #5 OR #6 OR #7 | 78253 |
| #9 | MeSH descriptor: [Rehabilitation, Vocational] explode all trees | 426 |
| #10 | MeSH descriptor: [Occupational Therapy] explode all trees | 700 |
| #11 | MeSH descriptor: [Employment, Supported] explode all trees | 120 |
| #12 | (Individual placement and support):ti,ab,kw | 250 |
| #13 | (individual placement):ti,ab,kw | 623 |
| #14 | (IPS):ti,ab,kw | 552 |
| #15 | (job coaching):ti,ab,kw | 36 |
| #16 | (supported employment):ti,ab,kw | 374 |
| #17 | #9 OR #10 OR #11 OR #12 OR #13 OR #14 OR #15 OR #16 | 2294 |
| #18 | MeSH descriptor: [Employment] explode all trees | 1775 |
| #19 | MeSH descriptor: [Work] explode all trees | 988 |
| #20 | (competitive employment):ti,ab,kw | 209 |
| #21 | (competitive work):ti,ab,kw | 298 |
| #22 | (competitive job):ti,ab,kw | 106 |
| #23 | (paid employment):ti,ab,kw | 182 |
| #24 | (paid work):ti,ab,kw | 409 |
| #25 | (paid job):ti,ab,kw | 101 |
| #26 | #18 OR #19 OR #20 OR #21 OR #22 OR #23 OR #24 OR #25 | 3159 |
| #27 | #8 AND #16 AND #25 with Publication Year from 1990 to 2019, in Trials | 243 |

**Supplementary materials 2. Extracted data from included studies**

1. Study details: Study name, number of publications, publication year, country, number of sites the study is executed in, the setting in which IPS is implemented and the trial registration number.

2. Participant characteristics: Number of recruited and randomized participants at baseline, drop-out rate, primary diagnosis, secondary or comorbid diagnosis, percentage female, age at onset, duration of illness, duration of untreated illness, ethnicity, marital status, living situation, education level, employment history, proportion that received benefits, baseline level of symptoms and functioning, medication use and the number or duration of previous hospitalizations.

3. Treatment variables: Intervention name, type of intervention (experimental vs control), duration of intervention, number of sessions, type of control group and fidelity assessment.

4. Outcomes: outcome name, outcome source, outcome type (continuous or categorical), direction of outcome (higher or lower score indicates favorable outcomes), assessment period, assessment method (i.e. self-report, clinician rated or based on registry data) and raw study outcomes of each outcome.

5. Study design and quality: method of randomization, blinding of outcome assessment, investigator, participants and employment specialists, selective outcome reporting, analysis method (intention to treat or per protocol) and method of handling missing data.

**Supplementary materials 3. Funnel plots**

**
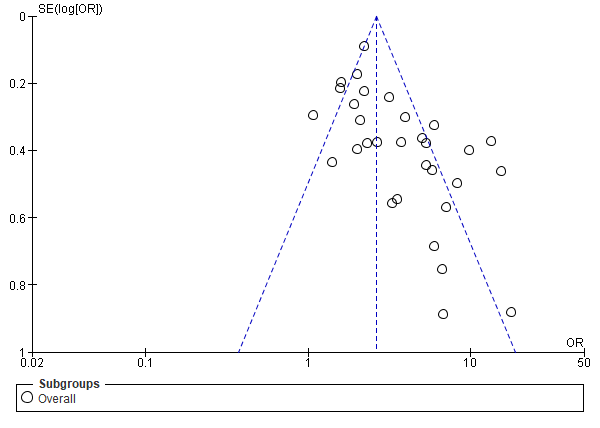
Employment rate**

**
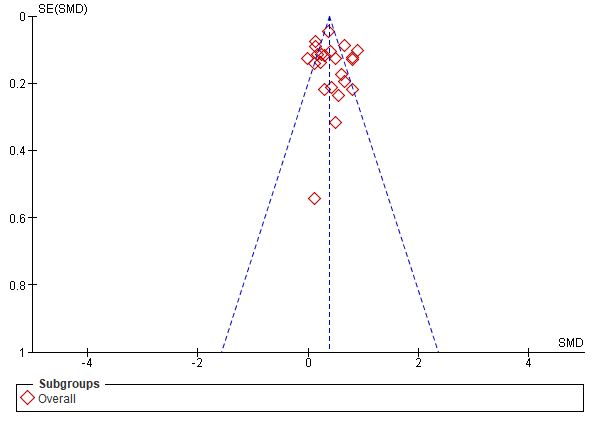
Job duration**

**
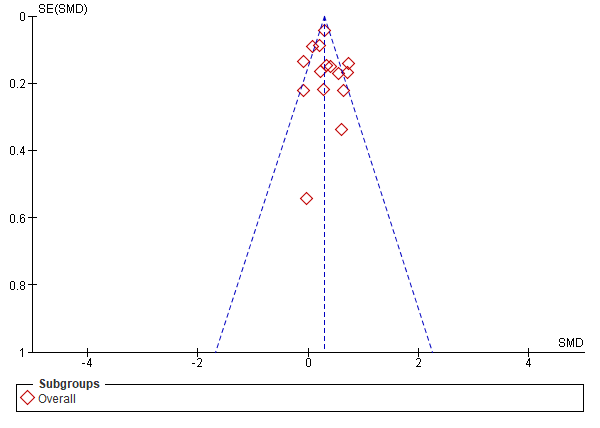
Wages**
